# Gut-derived hyodeoxycholate reprograms the spleen–eye immunometabolic axis to suppress autoimmune uveitis

**DOI:** 10.1038/s41418-026-01696-8

**Published:** 2026-03-11

**Authors:** Yitao Li, Weijia Zheng, Jiao Ma, Lu Liu, Xintong Yang, Junliang Kuang, Nickie Chan, Chengqiang Wang, Yang Li, Aihua Zhao, Ruonan Wang, Xiaojiao Zheng, Gerry Melino, Aiping Lu, Xiaolu Yang, Wei Jia

**Affiliations:** 1 School of Chinese Medicine, Hong Kong Baptist University, Hong Kong, China; 2 Department of Pharmacology and Pharmacy, The University of Hong Kong, Hong Kong, China; 3 Center for Translational Medicine and Shanghai Key Laboratory of Diabetes Mellitus, Shanghai Sixth People’s Hospital Affiliated to Shanghai Jiao Tong University School of Medicine, Shanghai, China; 4 Department of Ophthalmology, Shanghai General Hospital, Shanghai Jiao Tong University School of Medicine, Shanghai, China; 5 National Clinical Research Center for Eye Diseases, Shanghai General Hospital, Shanghai Jiao Tong University School of Medicine, Shanghai, China; 6 Department of Experimental Medicine, University of Rome “Tor Vergata”, Rome, Italy

**Keywords:** Metabolomics, Immunological disorders

## Abstract

Autoimmune uveitis (AU) lacks targeted therapies beyond immunosuppression. We identified hyodeoxycholate (HDCA), a gut-derived secondary bile acid, as a key immunometabolic regulator in AU. Metabolomics revealed systemic depletion of HDCA and oleic acid (C18:1n9) in AU patients and experimental AU (EAU) mice, correlating with disease severity. HDCA administration effectively attenuated EAU by reducing pro-inflammatory cytokines (IL-1β, IL-6, and TNF-α) and elevating IL-10. Mechanistically, HDCA inhibits Farnesoid X Receptor in splenic red pulp macrophages, activating SREBP1c-dependent fatty acid synthase, which enhances oleic acid production. Systemic oleic acid suppresses ocular Th17 responses and promotes M2 macrophage polarization, enhancing anti-inflammatory immunity. These findings define a spleen-to-eye immunometabolic axis driven by HDCA-mediated macrophage reprogramming, positioning HDCA as a promising therapeutic for AU.

**Figure:**
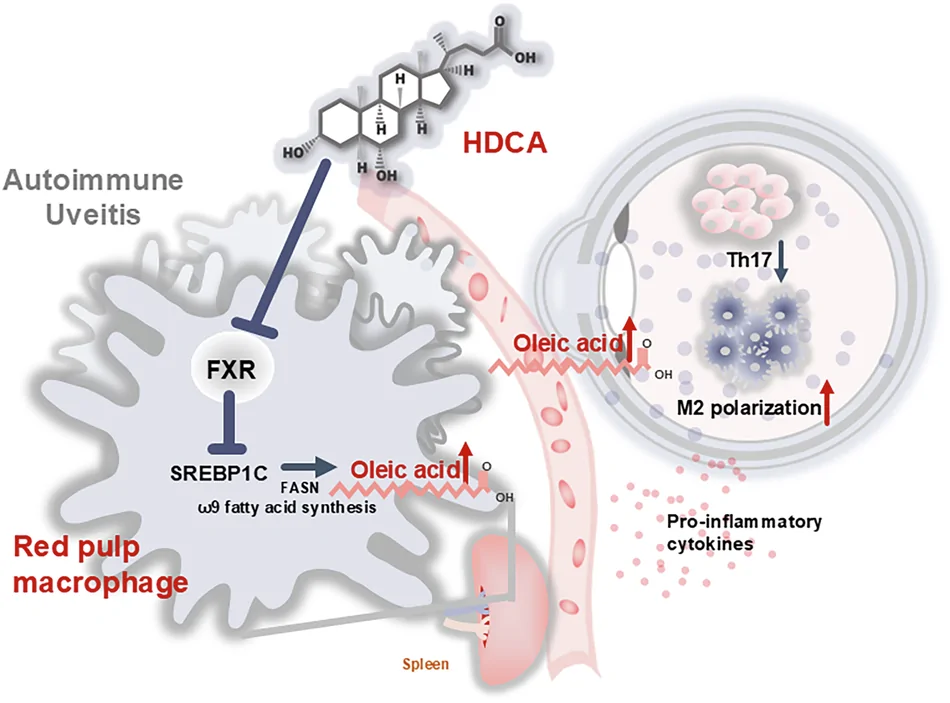

## Introduction

Autoimmune diseases, such as autoimmune uveitis (AU), are characterized by chronic inflammation and immune dysfunction, presenting significant treatment challenges due to complex interactions between immune dysregulation and tissue damage. Traditional treatment primarily involves immunosuppression, typically glucocorticoid therapy [1, 2], which often leads to significant side effects, including femoral head necrosis, abnormal blood sugar levels, and abdominal obesity [3]. Recent advancements in biological agents, including interleukin-17 (IL-17) and interleukin-6 (IL-6) receptor monoclonal antibodies, show promise in treatment [4]. However, their high cost may limit accessibility for many patients. Given these challenges, it is imperative to search for new treatment options that offer effective relief, minimize side effects, and are more cost-effective for broader patient access.

The eye possesses a remarkable immune privilege distinguished by the activation of sophisticated immunosuppressive biochemical pathways that regulate its interactions with the immune system [5]. This unique privilege is maintained by intricate mechanisms within immune organs, such as the spleen. Within these immune organs, macrophages are essential components of the innate immune system. They can be classified into two primary subtypes: tissue-resident macrophages (TRMs) and monocyte-derived circulating macrophages [6, 7]. TRMs constitute a diverse population distributed across various organs, each with distinct functions tailored to their specific micro-environments. Among them, antigen-presenting F4/80^+^ macrophages, particularly the splenic TRMs red pulp macrophages (RPMs), play a pivotal role in promoting immune tolerance [8, 9]. The absence of these cells disrupts immune tolerance, impairing the generation of regulatory T cells upon antigen exposure. This failure in immune regulation predisposes the eye to autoimmune attack [10]. The breakdown in ocular immune privilege further increases susceptibility to AU [11], with autoreactive immune cells, particularly expansion of pathogenic Th17 cells and M1-type macrophages, contributing to disease progression [12–14].

Hyodeoxycholate (HDCA), an endogenous bile acid that is abundant in pig bile but present only in trace amounts in humans, has emerged as a promising therapeutic molecule [15–18]. Our group previously established HDCA’s dual function in enhancing GLP-1 secretion to regulate glucose metabolism [15, 16] and stimulating unsaturated fatty acid (UFA) production, indicating its potential for treating non-alcoholic fatty liver disease [17]. Concurrently, HDCA exhibits immunomodulatory capacity through competitive LPS inhibition at TLR4/MD-2, driving M2 polarization while suppressing inflammation and protecting against sepsis [18]. In the current investigation, we disclose that HDCA can inhibit FXR in splenic RPMs, triggering SREBP1c-dependent oleic acid synthesis. RPM-derived oleic acid is rapidly secreted into circulation and accumulates systemically, suppressing ocular Th17 responses while promoting M2 polarization and anti-inflammatory activity, thereby alleviating EAU pathology. The se findings demonstrate that HDCA resolves AU by reprogramming RPM metabolism, positioning HDCA as a promising therapeutic candidate for AU.

## Results

### Cross-species AU metabolomics reveals HDCA and oleic acid deficits

**Fig. 1:**
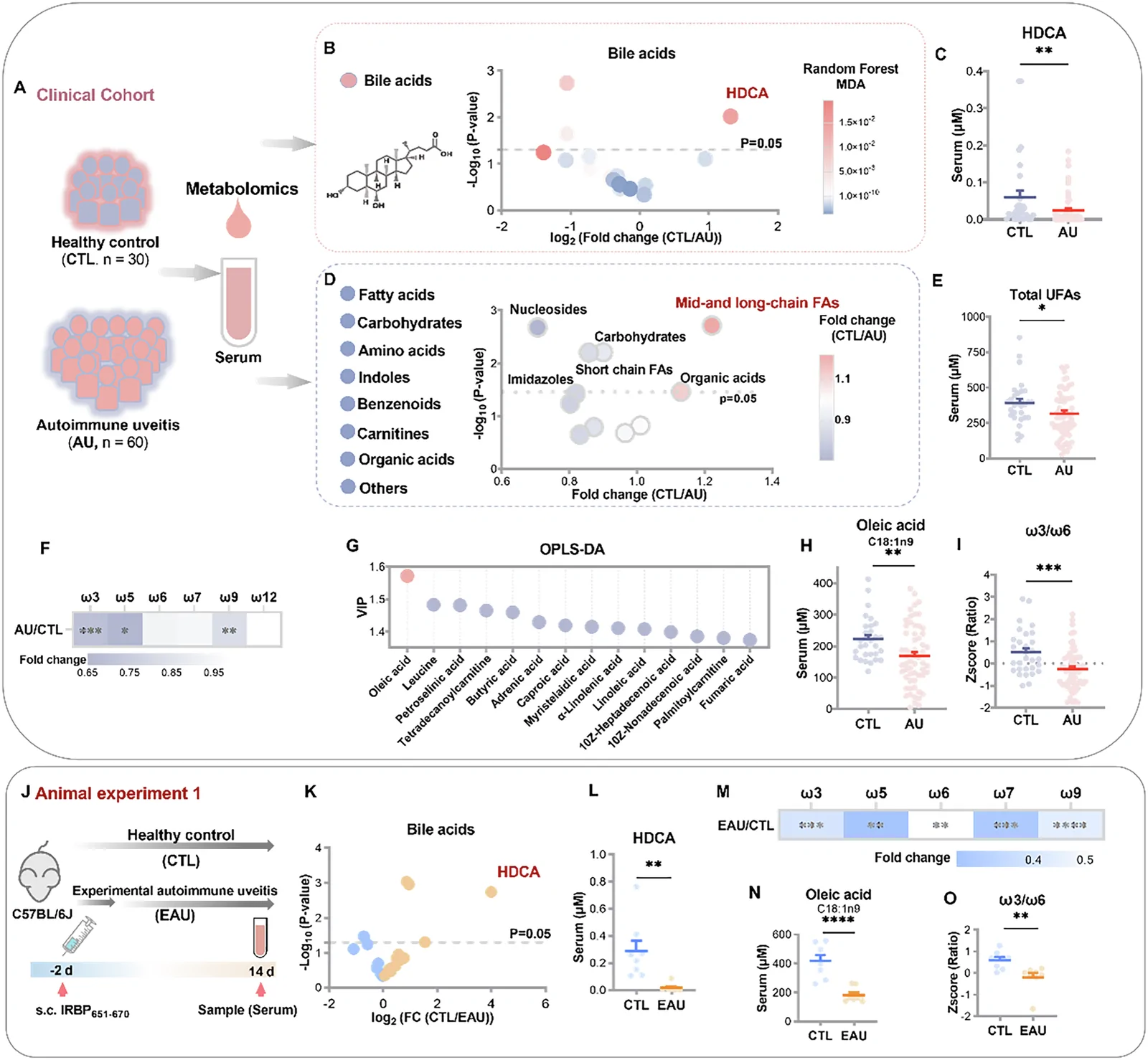
Cross-species AU metabolomics reveals HDCA and oleic acid deficits. **A** Grouping of the clinical cohort, including healthy controls (CTL, *n* = 30) and patients with autoimmune uveitis (AU, *n* = 60). **B** Volcano plot showing differential serum bile acid levels in the cohort, with Mean Decrease Accuracy (MDA) values from random forest analysis. **C** Serum HDCA levels in AU vs. CTL cohorts. **D** Serum metabolic profiling analysis, including amino acids, fatty acids, carnitines, carbohydrates, indoles, organic acids, phenylpropanoic acids, benzenoids, pyridines, nucleosides, and imidazoles. **E** Serum unsaturated fatty acid (UFA) levels in AU vs. CTL cohorts. **F** Fold changes of UFAs of AU to CTL. **G** OPLS-DA analysis based on serum metabolic profiling in (**D**). **H** Serum oleic acid levels in AU vs. CTL cohorts. **I** Serum ω3/ω6 fatty acid ratios in AU vs. CTL cohorts. **J** Experimental autoimmune uveitis (EAU) mouse model was established in female C57BL/6J mice via immunization with IRBP651-670 peptide in complete Freund’s adjuvant (CFA), along with lipopolysaccharide (LPS) and pertussis toxin (PTX) administration. Age-matched healthy mice served as controls (*n* = 8 per group). **K** Volcano plot showing differential serum bile acid levels in mice, with MDA values of random forest analysis. **L** Serum HDCA levels in EAU vs. control mice. **M** Fold changes of UFAs of EAU to control mice. **N** Serum oleic acid levels in EAU vs. control mice. **O** Serum ω3/ω6 fatty acid ratios in EAU vs. control mice.

Our quantitative metabolomic profiling of serum samples from 90 participants, including 60 AU patients and 30 healthy controls (CTL) at Shanghai General Hospital, identified significant metabolic perturbations associated with autoimmune uveitis (Fig. 1A and Table S1). The analysis first revealed distinct alterations in bile acid metabolism, with HDCA emerging as the most significantly depleted bile acid species in AU patients compared to the CTL group (Figs. 1B, C and S1A).

Concurrently, metabolomic profiling including amino acids, fatty acids, carnitines, carbohydrates, indoles, organic acids, phenylpropanoic acids, benzenoids, pyridines, nucleosides, and Imidazoles revealed marked dysregulation in mid- and long-chain fatty acid metabolism, particularly a pronounced reduction in total unsaturated fatty acids (UFAs; Figs. 1D, E and S1B). Further analysis of serum UFA levels showed significant decreases in ω3, ω5, and ω9 species compared to the CTL group (Fig. 1F). While ω3 species including α-linolenic, eicosapentaenoic, and docosahexaenoic acids showed the most significant alterations overall, oleic acid (C18:1n9), an ω9 fatty acid, exhibited the most pronounced reduction among individual UFAs in AU patients (Fig. 1G, H). Notably, the ratios of serum ω3 fatty acid levels to ω6 species (including γ-linolenic, arachidonic, and dihomo-γ-linolenic acids) known to correlate with inflammation severity [19], was significantly reduced in AU patients herein (Fig. 1I).

Similar metabolic perturbations were observed in the experimental autoimmune uveitis (EAU) mouse model (Fig. 1J). EAU mice faithfully reproduced the HDCA deficiency observed in patients, along with characteristic UFA alterations including reduced serum oleic acid and diminished ω3/ω6 fatty acid ratios (Figs. 1K–O and S1C, D). These cross-species findings highlight HDCA and oleic acid depletion as conserved metabolic hallmarks of uveitis-associated inflammation, nominating them as potential diagnostic biomarkers and mechanistic targets.

### HDCA alleviates autoimmune uveitis through suppressing inflammation

While both HDCA and oleic acid show promise as therapeutic targets for AU, systemic administration of free fatty acids may cause undesirable metabolic disturbances that could potentially lead to other pathological conditions [20, 21]. This prompted us to investigate HDCA as a potentially safer pharmacological alternative.

**Fig. 2:**
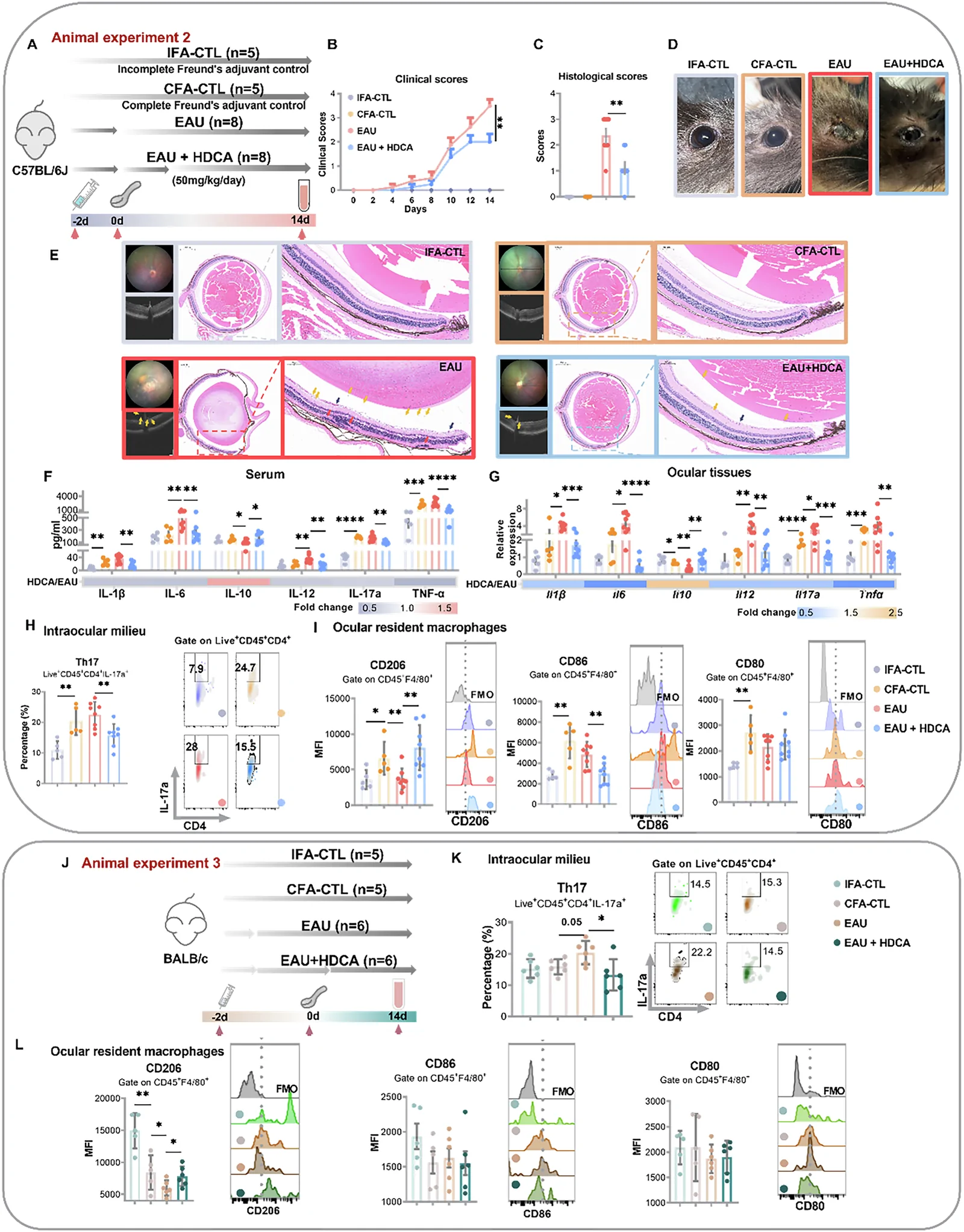
HDCA alleviates AU through suppressing inflammation. **A** C57BL/6J mice were divided into four groups: IFA control (incomplete Freund’s adjuvant only; *n* = 5), CFA control (complete Freund’s adjuvant only; *n* = 5), EAU model (*n* = 8), and EAU model with oral HDCA at 50 mg/kg/day (*n* = 8) for 14 days. **B** Clinical scores (0–4 scale) of the four groups, including IFA control, CFA control, EAU, and EAU with HDCA treatment (50 mg/kg/day). **C** Histological scores (0-3 scale) of the four groups. **D** Representative images show ocular changes in the four; groups. **E** Hematoxylin and eosin (H&E) staining images of eye sections on day 14 post-immunization exhibit the retinal folds (red arrows), neovascularization (blue arrows), and inflammatory cell infiltration (yellow arrows) across the four groups. **F** Serum levels of pro- and anti-inflammatory cytokines, along with a heatmap of the HDCA-treated to EAU group ratio. **G** Relative expression of *Il1β*, *Il6*, *Il10, Il12, Il17* and *Tnfα* in the retina at 14 days. **H** Flow cytometry analysis shows the percentages of Th17 cells (CD45+CD4+IL-17a+) among live cells across the four groups. **I** Flow cytometry analysis shows the mean fluorescence intensity (MFI) of CD206, CD86, and CD80 on ocular macrophages (CD45^+^F4/80^+^) across the four groups. **J** BALB/c mice were divided into four groups (*n* = 5 for control groups; *n* = 6 for treatment groups): IFA-control, CFA-control, EAU model, and EAU model with oral HDCA at 50 mg/kg/day for 14 days. **K** Flow cytometry analysis shows the percentages of Th17 cells across the four groups. **L** Flow cytometry analysis shows the MFI of CD206, CD86, and CD80 on ocular macrophages across the four groups.

To evaluate HDCA’s therapeutic potential for AU, we established an experimental autoimmune uveitis (EAU) model in C57BL/6J mice by injecting IRBP. The study comprised four experimental groups: incomplete Freund’s adjuvant (IFA) control group, complete Freund’s adjuvant (CFA) control group (both control groups included to eliminate potential immunostimulatory effects from inactivated *Mycobacterium tuberculosis*), EAU model group, EAU model group administered HDCA orally at 50 mg/kg/day, a dosage previously established as safe [15, 17, 18] (Fig. 2A). Uveitis severity was evaluated using a clinical score (0–4 scale; 0: no disease, 4: severe inflammation) while a histological score (0–3 scale) assessing immune infiltration, retinal folds, and other ocular damage. This systematic approach demonstrated that HDCA treatment produced significant clinical improvement by day 14, with treated EAU mice demonstrating both reduced clinical scores and improved histopathological outcomes compared to untreated controls (Fig. 2B, C). Notably, HDCA-treated EAU mice developed characteristic white pearlescent secretions and exhibited substantial ocular tissue remodeling (Fig. 2D). In addition, fundus imaging also revealed alleviated optic disc inflammation and reduced retinal tissue damage (white arrows) in HDCA-treated mice. Optical coherence tomography (OCT) and histopathological analysis further confirmed the therapeutic efficacy of HDCA, demonstrating decreased inflammatory infiltration (monocytes, lymphocytes, and erythrocytes; orange arrows), suppressed retinal folding (red arrows), diminished neovascularization (blue arrows), and overall attenuation of disease severity (Fig. 2E).

At the molecular level, cytokine profiling at the experimental endpoint showed HDCA-mediated modulation of serum pro-inflammatory cytokines, including the significant reductions in IL-1β, IL-6, IL-12, IL-17a, and TNF-α and corresponding increases in ocular pro-inflammatory IL-10 expression (Fig. 2F, G). To further elucidate HDCA’s immunomodulatory properties, we investigated two critical immune cell populations implicated in AU pathogenesis: adaptive Th17 cells [12, 22] and innate macrophages [23]. Results revealed that HDCA treatment significantly reduced ocular Th17 cell differentiation while promoting macrophage polarization toward an M2 phenotype, as evidenced by elevated CD206 expression with diminished M1 markers CD86 and CD80 in EAU mice (Fig. 2H, I; Gate strategies, Fig. S2A, B).

Subsequently, HDCA’s therapeutic efficacy extended to the typically EAU-resistant BALB/c strain, which exhibits milder baseline disease, where treatment induced significant improvements in clinical and histological scores, while systemically attenuating pro-inflammatory cytokine release (Figs. 2J and S2C–F). Concurrently, in the BALB/c strain, HDCA treatment suppressed ocular Th17 cell differentiation and promoted M2 macrophage (CD206^+^) polarization (Fig. 2K, L). These cross-strain findings demonstrate HDCA’s capacity for broad inflammation suppression in AU.

### HDCA reprograms splenic red pulp macrophages to constrain autoimmunity

**Fig. 3:**
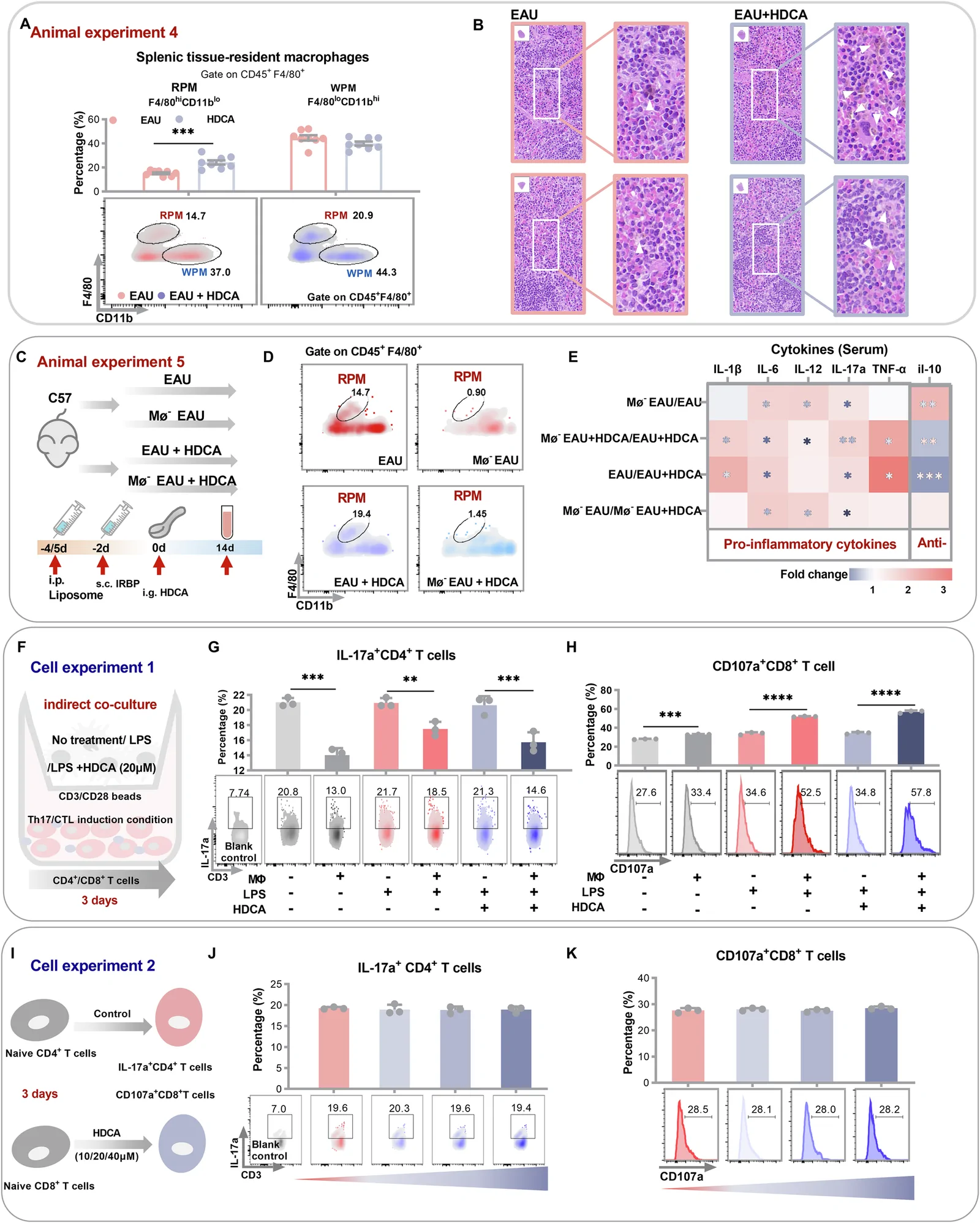
HDCA reprograms splenic red pulp macrophages to constrain autoimmunity. **A** C57BL/6J mice were divided into two groups: EAU model, and EAU model with HDCA treatment at 50 mg/kg/day for 14 days. The splenic residue macrophages, including red pulp macrophages (CD45^+^F4/80^hi^CD11b^lo^) and white pulp macrophages (CD45^+^F4/80^lo^CD11b^hi^), were analyzed (*n* = 8 per group). **B** Representative H&E-stained images show hemosiderin^+^ macrophages (white arrows) within the red pulp zones of EAU and HDCA-treated EAU mice. **C** C57BL/6J mice were divided into four groups: EAU model, EAU model with HDCA treatment (50 mg/kg/day), macrophage-depleted EAU model (Mø^-^EAU), and Mø^-^EAU model with HDCA treatment for 14 days (*n* = 4 per group). **D** Flow cytometry analysis of RPMs across the four groups. **E** Heatmap of fold changes in serum levels of pro- and anti-inflammatory cytokines. **F** Naïve CD4+ or CD8+ T cells were indirectly co-cultured with BMDMs with LPS, or LPS + HDCA (20 μM) under Th17 or cytotoxic T lymphocyte induction conditions for 72 h. **G** The percentages of Th17 cells (IL-17a^+^). **H** The percentages of cytotoxic T lymphocytes (CD107a^+^). **I** Naïve CD4+ T cells and BMDMs were cultured with varying concentrations of HDCA (10, 20, and 40 μM) Th17 or M1 induction conditions for 72 h (T cells) or 24 h (BMDMs), respectively. **J** The percentages of Th17 cells. **K** The percentages of cytotoxic T lymphocytes (CD107a^+^).

Evidence suggests that orally administered HDCA exhibits immunomodulatory effects in mice [18] and accumulates at high concentrations in the spleen [24], a finding further supported by our pharmacokinetic data (Fig. S2G). To investigate the immunomodulatory effects of HDCA, we analyzed splenic immune cell populations in EAU and HDCA-treated mice. Data demonstrated that HDCA selectively expanded red pulp macrophages (RPMs; CD45⁺F4/80ʰ^i^CD11bˡᵒ) but did not significantly affect other immune subsets, including white pulp macrophages (WPMs, CD45^+^ F4/80^low^ CD11b^hi^), natural killer cells (CD3^-^ Nkp46^+^), γδ^+^ T cells (CD3^+^ γδ^+^), CD4^+^ T cells (CD3^+^ CD4^+^), and CD8^+^ T cells (CD3^+^ CD8^+^) (Figs. 3A and S3A–C). Histological analysis further confirmed RPM expansion, with HDCA-treated spleens exhibiting abundant hemosiderin⁺ macrophages (white arrows) in red pulp zones (Fig. 3B). Next, to validate the role of RPMs in EAU, we generated macrophage-depleted (Mø^-^) EAU mice using clodronate liposomes, as verified by flow cytometry showing RPM depletion exceeding 90% (Fig. 3C, D). Following 14 days of HDCA treatment, we performed reciprocal verification by comparing serum cytokine profiles, including IL-1β, IL-6, IL-17a, IL-12, and TNF-α, and IL-10 in EAU mice, macrophage-depleted EAU mice and controls. Results revealed that HDCA failed to downregulate serum pro-inflammatory factors or upregulate anti-inflammatory cytokines in macrophage-depleted mice (Fig. 3E).

To investigate the role of RPMs in HDCA’s systemic immunomodulation of distal tissues, we established an indirect co-culture system. Immune cells, including CD4^+^ T cells under Th17 induction conditions and CD8^+^ T cells under cytotoxic T lymphocyte (CD107a^+^) induction conditions, were cultured with LPS- or HDCA-treated (20 μM) bone marrow-derived macrophages (BMDMs) for 72 h (Fig. 3F). Flow cytometry analysis revealed that macrophages potently suppressed Th17 and promoted CD107a^+^CD8^+^ T cell differentiation in vitro (Fig. 3G, H). Next, we examined HDCA’s direct effects on resident immune cell differentiation by culturing naïve CD4^+^ T cells, CD8 + T cells or BMDMs with HDCA alone under identical stimulating conditions. Notably, HDCA treatment exhibited no direct inhibitory effect on CD4^+^ or CD8^+^ T cell differentiation, indicating that its immunomodulatory effects are macrophage-dependent (Fig. 3I–K). Together, these results demonstrate that HDCA requires RPMs to mediate its distal immunomodulatory effects.

### HDCA facilitates RPM-dependent oleic acid homeostasis

Our metabolomic profiling of autoimmune uveitis (AU) patients and experimental AU (EAU) mice identified oleic acid as another key metabolite significantly reduced in circulation alongside HDCA (Fig. 1). We therefore examined whether HDCA treatment could potentially restore circulating oleic acid levels in EAU mice and explored their relationship with RPM populations.

**Fig. 4:**
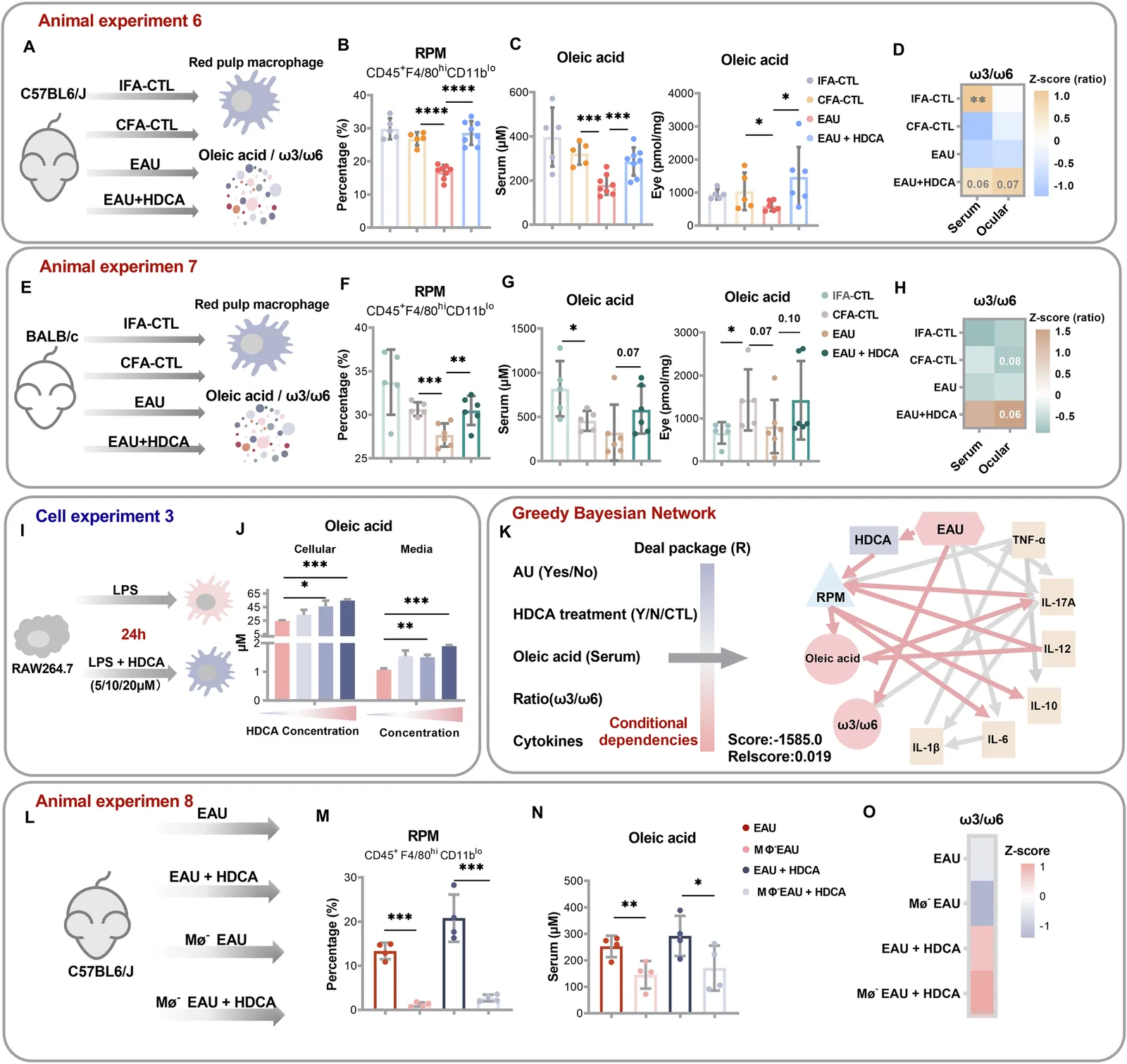
HDCA facilitates RPM-dependent oleic acid homeostasis. **A** C57BL/6J mice were divided into four groups: IFA-control, CFA-control, EAU models, and EAU models with HDCA treatment at 50 mg/kg/day for 14 days (*n* = 5 for control groups; *n* = 8 for treatment groups). **B** The percentages of RPMs. **C** Serum and ocular oleic acid levels (*n* = 6 for treatment groups). **D** Serum and ocular ω3/ω6 fatty acid ratios. **E** BALB/c mice were divided into four groups: IFA-control, CFA-control, EAU models, and EAU models with HDCA treatment (*n* = 5 for control groups; *n* = 6 for treatment groups). RPMs and oleic acid levels, as well as ω3/ω6 fatty acid ratios in serum and ocular tissues, were analyzed. **F** The percentages of RPMs. **G** Serum and ocular oleic acid levels. **H** Serum and ocular ω3/ω6 fatty acid ratios. **I** RAW264.7 cells were cultured with varying concentrations of HDCA (5, 10, and 20 μM) under M1 induction conditions for 24 h. **J** Intracellular and extracellular oleic acid levels. **K** Greedy Bayesian Network (GBN) was constructed incorporating the following variables: disease status, HDCA treatment, fatty acids, including the ω3/ω6 fatty acid ratios and oleic acid levels, relative proportions of immune cells (RPMs), and pro- and anti-inflammatory cytokines (IL-1β, IL-6, IL-12p70, IL-17A, IL-10, and TNF-α). The network was constructed by using the “Deal” package in R software, which employs a greedy search algorithm to iteratively score candidate structures based on the Bayesian Information Criterion (BIC) and posterior probabilities. **L** C57BL/6J mice were divided into four groups: EAU model, EAU model with HDCA treatment (50 mg/kg/day), macrophage-depleted EAU model (Mø^-^EAU), and Mø^-^EAU model with HDCA treatment for 14 days (*n* = 4 per group). **M** The percentages of RPMs. **N** Serum oleic acid levels. **O** Serum ω3/ω6 fatty acid ratios.

First, we performed animal studies using both C57BL/6J and BALB/c mouse strains, conducting flow cytometric analysis of RPM populations and serum metabolomic profiling of UFAs across four experimental groups, including EAU mice, HDCA-treated EAU mice, IFA controls, and CFA controls. Following 14-day HDCA treatment, we observed a coordinated immunometabolic response characterized by significant expansion of RPMs alongside obvious restoration of serum oleic acid levels in treated mice (Fig. 4A–F). In contrast to C57BL/6J mice, BALB/c mice exhibit elevated baseline levels of RPMs and circulating oleic acid, correlated with their enhanced resistance to EAU induction [25] (Fig. 4G, H). Notably, HDCA treatment significantly increased the ω3/ω6 fatty acid ratios in both strains, indicative of an anti-inflammatory metabolic shift.

Next, we conducted in vitro experiments using RAW264.7 cells to investigate whether HDCA stimulates oleic acid production in macrophages (Fig. 4I). Results revealed that HDCA treatment markedly elevated oleic acid levels in inter- and intracellular compartments, demonstrating that HDCA not only systemically restores oleic acid in EAU but also directly enhances its biosynthesis in macrophages (Figs. 4J and S4). Furthermore, we employed Bayesian network analysis to establish causal relationships within the system. The modeling revealed that HDCA-induced RPM reprogramming initiates a metabolic cascade wherein enhanced oleic acid metabolism directly regulates key inflammatory mediators (Fig. 4K). Conversely, in macrophage-depleted C57BL/6J EAU mice (Mø^-^ model establishment was described before), HDCA treatment (50 mg/kg) failed to restore oleic acid levels or alter the ω3/ω6 fatty acid ratios (Fig. 4L–O).

Together, our data indicate that HDCA resolves inflammation through RPM-dependent metabolic rewiring, with oleic acid elevation serving as both a key effector molecule and biomarker of macrophage reprogramming.

### RPM-derived oleic acid as an anti-inflammatory resolution mediator

**Fig. 5:**
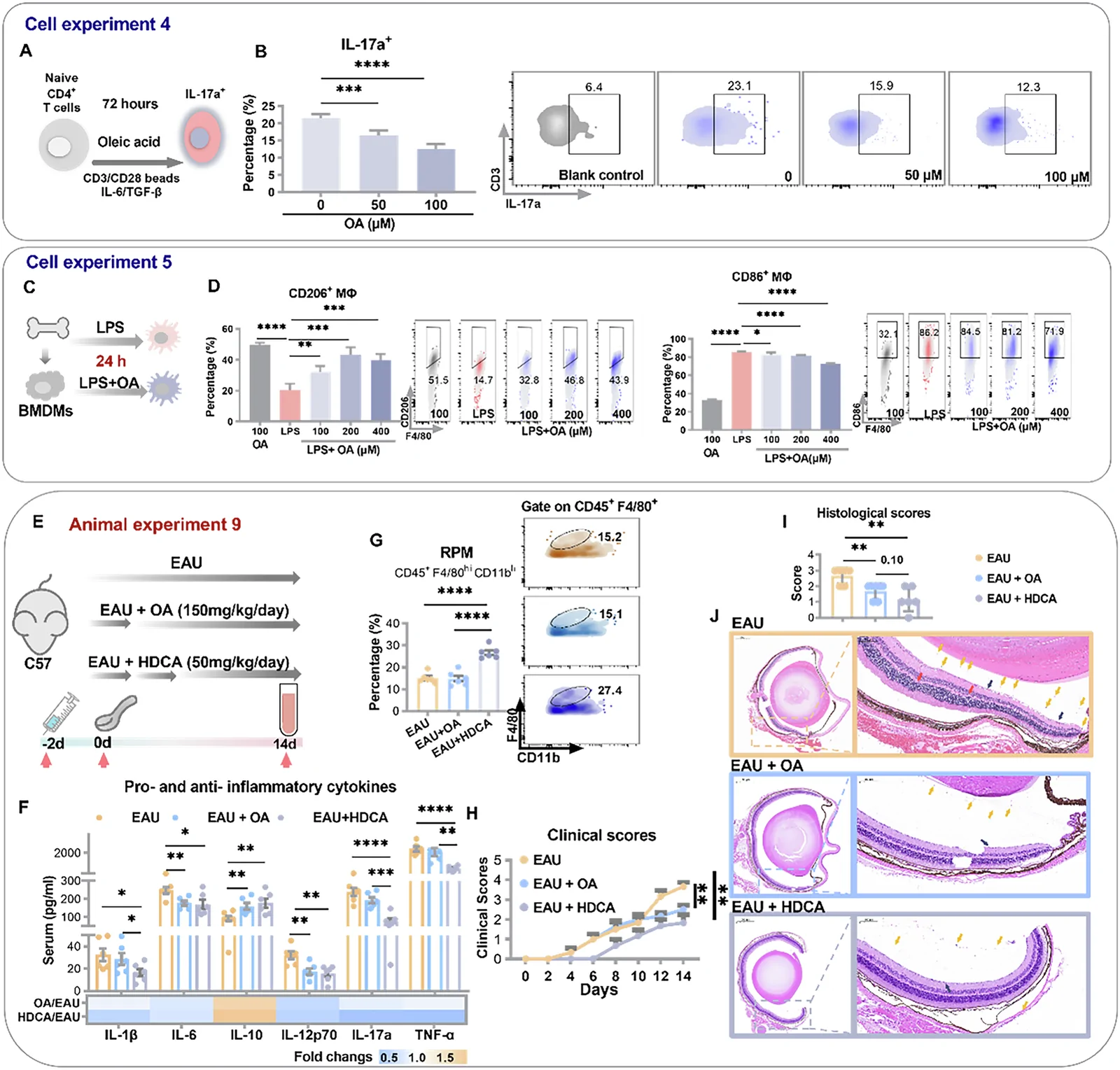
RPM-derived oleic acid as an anti-inflammatory resolution mediator. **A** Naïve CD4 T cells were cultured with varying concentrations of oleic acid (50 and 100 μM) under Th17 induction conditions for 72 h (*n* = 3 per group). **B** The percentages of Th17 cells (IL-17a^+^). **C** BMDMs were cultured with varying concentrations of oleic acid (50 and 100 μM) under M1 induction conditions for 24 h (*n* = 3 per group). **D** The percentages of CD206^+^ macrophages (M2) and CD86^+^ macrophages (M1). **E** C57BL/6J mice were divided into three groups: EAU model, EAU model with oleic acid at 150 mg/kg/day, EAU model with HDCA treatment at 50 mg/kg/day for 14 days (*n* = 6 per group). **F** Serum levels of pro-/ anti-inflammatory cytokines. **G** Flow cytometry analysis shows the percentages of RPMs across the three groups. **H** Clinical scores of the three groups. **I** Histological scores of the three groups. **J** Hematoxylin and eosin (H&E) staining images of eye sections on day 14 post-immunization exhibit the retinal folds (red arrows), neovascularization (blue arrows), and inflammatory cell infiltration (yellow arrows) across the three groups.

We first examined the immunomodulatory effects of RPM-derived oleic acid on key immune cell populations through in vitro experiments to elucidate its role in EAU. Specifically in vitro, we stimulated naïve CD4^+^ T cells to differentiate into Th17 cells and treated them with oleic acid (50 and 100 μM). Consistent with our hypothesis, the results demonstrated dose-dependent suppression of Th17 differentiation (IL-17a^+^) by oleic acid (Fig. 5A, B). In another parallel experiment, LPS-stimulated BMDMs were cultured with oleic acid (100, 200, and 400 μM), and results revealed a striking enhancement of M2-type macrophages (CD206^+^) and reduction of M1-type CD86^+^ macrophage (Figs. 5C, D and S5). The in vitro experimental data suggest that oleic acid exerts anti-inflammatory potential, as evidenced by its dual capacity to suppress pathogenic Th17 cell differentiation and promote M2 macrophage polarization.

To validate these findings in vivo, we evaluated the therapeutic potential of oleic acid (150 mg/kg/day) and HDCA (50 mg/kg/day for 14 days) in EAU mice (Fig. 5E). Intriguingly, results indicate that HDCA and oleic acid treatment both possess anti-inflammatory properties, as evidenced by significantly reduced serum levels of pro-inflammatory cytokines IL-6, IL-12, and TNF-α while elevating anti-inflammatory IL-10. However, in contrast to HDCA, oleic acid exhibited markedly inferior therapeutic efficacy (Fig. 5F). Mechanistically, flow cytometric analysis revealed a critical difference that oleic acid treatment failed to elevate RPM levels (Fig. 5G). This observation suggests that while oleic acid can directly suppress Th17 differentiation and promote M2 polarization, it lacks HDCA’s capacity for systemic immunomodulation through RPM-mediated pathways.

Supporting this conclusion, comprehensive ocular evaluations incorporating clinical and histopathological scoring systems, along with H&E-stained tissue analysis, uniformly demonstrated that oleic acid administration only modestly attenuated EAU progression, with significantly weaker effects than HDCA (Fig. 5H–J). Collectively, our results establish that RPM-derived oleic acid is a key mediator of HDCA’s anti-inflammatory effects, while demonstrating that oleic acid alone cannot regulate RPM populations or achieve comparable therapeutic outcomes in EAU.

### HDCA promotes oleic acid synthesis in RPMs by modulating SREBP1c

**Fig. 6:**
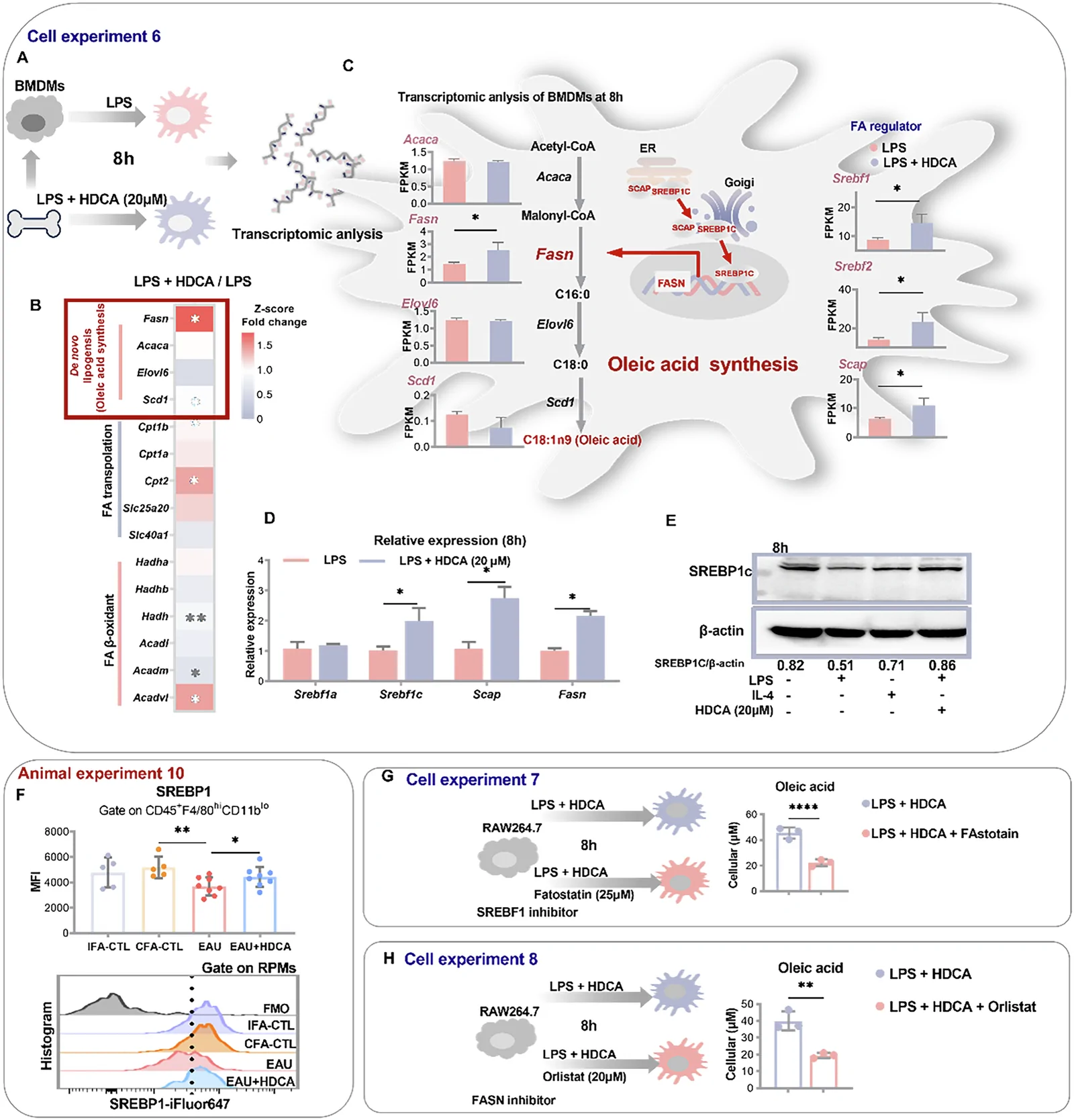
HDCA promotes oleic acid synthesis in RPMs by modulating SREBP1c. **A** In this cell experiment, BMDMs were treated with LPS or LPS + HDCA (20 μM) for 8 h, and the transcriptomics analysis was conducted. **B** Transcriptomic analysis of fatty acid metabolism pathways including oleic acid synthesis, fatty acid transportation, and fatty acid β-oxidation. **C** Transcriptomic profiling of enzymes and regulatory factors associated with oleic acid biosynthesis. **D** Relative expression of *Srebf1a*, *Srebf1c*, *Scap*, and *Fasn* in LPS-treated BMDMs with or without HDCA treatment at 8 h (*n* = 3 per group). **E** Protein expression of SREBP1 in LPS-treated BMDMs with or without HDCA treatment at 8 h. **F** Flow cytometry analysis of SREBP1 expression in RPMs in IFA-CTL, CFA-CTL, EAU, and HDCA-treated EAU C57BL/6J mice (*n* = 5 for control groups; *n* = 8 for treatment groups). **G** RAW264.7 cells were co-stimulated with LPS and HDCA (20 μM) in the presence or absence of the SREBP1 inhibitor fatostatin (25 μM), and cellular oleic acid levels were quantified at 8 h (*n* = 3 per group). **H** RAW264.7 cells were co-stimulated with LPS and HDCA, in the presence or absence of the FASN inhibitor orlistat (20 μM) for 8 h, and the cellular levels of oleic acid levels are quantified (*n* = 3 per group).

To elucidate the mechanism underlying HDCA-mediated promotion of oleic acid production, we conducted cell experiments using LPS-stimulated BMDMs with or without HDCA (20 μM) for 8 h (Fig. 6A). Metabolomic analysis demonstrated that cellular oleic acid accumulation is driven primarily by lipogenic pathway upregulation (Figs. 4I, J and S4). Specifically, through activation of the master regulator *Srebf* family and its downstream target *Fasn,* which is critical for ω9 fatty acid synthesis, with all HDCA-treated groups exhibiting statistically significant induction of these genes (Fig. 6B, C). Notably, HDCA obviously upregulates *Srebf1c* expression while showing no significant effect on *Srebp1a* (Fig. 6D). These transcriptional alterations were corroborated at the translational level, as evidenced by western blot quantification of augmented SREBP1c expression (Fig. 6E; SI). Next, we conducted the parallel assessments of SREBP1c expression in RPMs isolated from C57BL/6J mice. Results revealed that HDCA administration elicited significant SREBP1c induction in mice, strikingly concordant with our in vitro observations (Fig. 6F).

To functionally characterize the roles of SREBP1c and FASN in HDCA-mediated oleic acid metabolism, we conducted an 8-h pharmacological inhibition of SREBP1c or FASN in RAW264.7 cells using their respective inhibitors. In parallel experiments, SREBP1c inhibition with fatostatin (25 μM) completely abolished HDCA-induced oleic acid accumulation (Fig. 6G), while FASN blockade with orlistat (20 μM) similarly prevented HDCA-mediated oleic acid production (Fig. 6H). The results showed that HDCA failed to upregulate oleic acid synthesis when these enzymes were inhibited, demonstrating that HDCA promotes oleic acid synthesis through sequential activation of SREBP1c and its downstream target FASN. Together, HDCA promotes RPM-derived oleic acid synthesis through activation of the SREBP1c-FASN axis.

### HDCA restores oleic acid biosynthesis through FXR-SREBP1c axis

To further investigate the molecular mechanisms underlying the interplay between immune activation and fatty acid metabolic reprogramming, we utilized Restricted Cubic Splines (RCS) to build receptor models related to oleic acid and Th17 among seven receptors, including *Fxr, Tgr5, Car, Vdr, Ahr, Rxr*, and *Ppar* (Fig. S6). For the oleic acid-related model, BMDMs were directly treated with HDCA and LPS, and the oleic acid model was constructed based on the relative expression of receptors and cellular levels of oleic acid. For the Th17-related model, BMDMs treated with LPS and different concentrations of HDCA were indirectly co-cultured with CD4 T cells under Th17 induction conditions, and the model was constructed between the percentage of Th17 and the relative expression of receptors. Among these receptors, FXR exhibited the strongest correlation with both immune responses and fatty acid metabolism. Spleen-targeted bile acid profiling in wild-type mice revealed a predominance of FXR-inhibiting over FXR-activating species, indicating tonic FXR suppression in RPM niches (Fig. S7A).

**Fig. 7:**
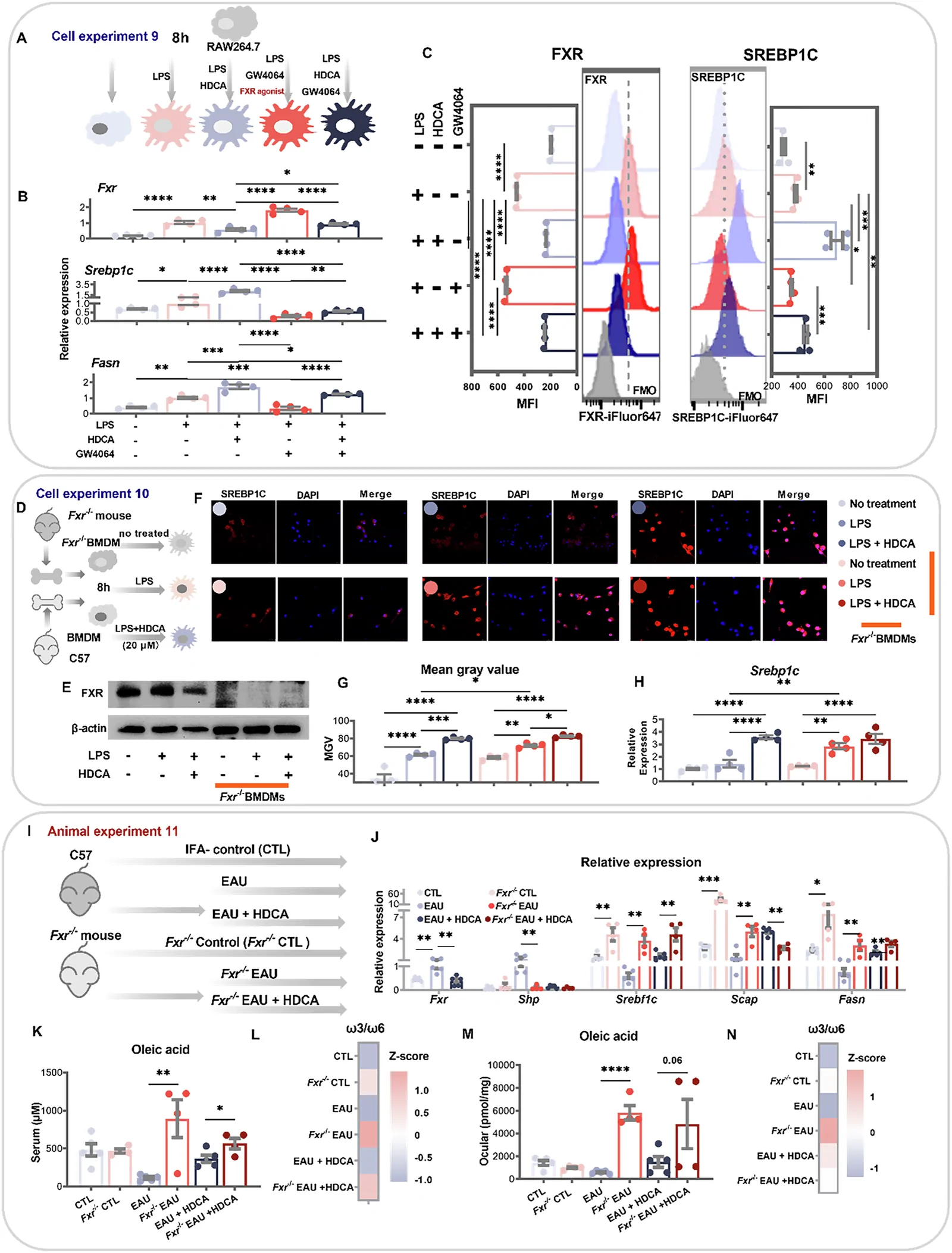
HDCA restores oleic acid biosynthesis through FXR-SREBP1c axis. **A** In this cell experiment, LPS-stimulated BMDMs were treated with or without GW4064 (10 μM) or HDCA (20 μM) for 8 h (*n* = 4 per group). **B** Relative expression of *Fxr*, *Srebf1c*, and *Fasn*. **C** Protein expression of FXR and SREBP1c. **D** In this cell experiment, *Fxr*^–/–^ BMDMs isolated from *Fxr*^–/–^ mice were stimulated by LPS and treated with or without HDCA (20 μM) for 8 h. **E** Protein expression of FXR. **F** Fluorescence immunoreactivity of SREBP1 (Red) with DAPI (Blue). **G** Mean gray value of fluorescence immunoreactivity. **H** Relative expression of *Srebf1c*. **I** C57BL/6J and *Fxr*^*–/–*^ C57BL/6J mice were separately divided into three groups: IFA control group, EAU model, and EAU model with HDCA treatment at 50 mg/kg/day for 14 days (*n* = 5 per group). **J** Splenic relative expression of *Fxr*, *Shp*, *scap*, *Srebf1c*, and *Fasn*. **K** Serum oleic acid levels. **L** Serum ω3/6 fatty acid ratios. **M** Ocular oleic acid levels. **N** Ocular ω3/6 fatty acid ratios.

To elucidate FXR’s role in HDCA-mediated regulation of oleic acid synthesis, we treated BMDMs with the FXR agonist GW4064 (10 μM) under LPS stimulation, with or without HDCA co-treatment (Fig. 7A). Quantitative PCR analysis demonstrated that HDCA treatment suppressed *Fxr* expression while upregulating key lipogenic genes including *Srebp1c* and *Fasn* associated with ω9 FA biosynthesis (Fig. 7B). Flowcytometry analysis further confirmed that HDCA treatment suppressed FXR expression while concomitantly upregulating SREBPc levels (Fig. 7C). Subsequently, we exposed *Fxr*^*−/−*^ C57BL/6J mouse-derived BMDMs to LPS stimulation, with parallel treatments of either 20 μM HDCA or vehicle control for 8 h (Fig. 7D). Western blot analysis confirmed the successful generation of *Fxr* ^−/−^ BMDMs, as evidenced by the absence of FXR protein expression (Fig. 7E; SI). Genetic ablation of *Fxr* significantly increased SREBP1 levels compared with wild-type BMDM controls (Fig. 7F–H), indicating that FXR exerts a constitutive repressive effect on lipogenic signaling, even under inflammatory conditions.

To validate these findings in vivo, wild-type and *Fxr*^*–/–*^ C57BL/6J mice were employed to establish the EAU model and administered either HDCA or vehicle control (Fig. 7I). Splenic flow cytometric evaluation demonstrated that relative to wild-type counterparts, *Fxr*^*–/–*^ animals displayed elevated RPM frequencies across all knockout cohorts (Fig. S7B). Simultaneously, *Fxr*^*–/–*^ mice displayed marked elevation in splenic expression of *Srebp1c*, *Scap* and *Fasn* mRNA, indicating that HDCA failed to promote oleic acid biosynthesis in *Fxr*-deficient animals (Fig. 7J). Furthermore, metabolomic profiling of serum and ocular tissues also revealed that *Fxr* ablation abolished HDCA’s ability to systemically enhance oleic acid production and ω3/ω6 fatty acid ratios (Fig. 7K–N).

Integrating genetic and metabolomic evidence demonstrates that HDCA restores oleic acid biosynthesis through FXR-dependent modulation of the SREBP1c pathway.

### HDCA ameliorates autoimmune uveitis through metabolic-immune crosstalk

We next investigated whether HDCA exerts therapeutic effects on ocular tissues in AU by modulating the FXR signaling immune-metabolic axis in RPMs.

**Fig. 8:**
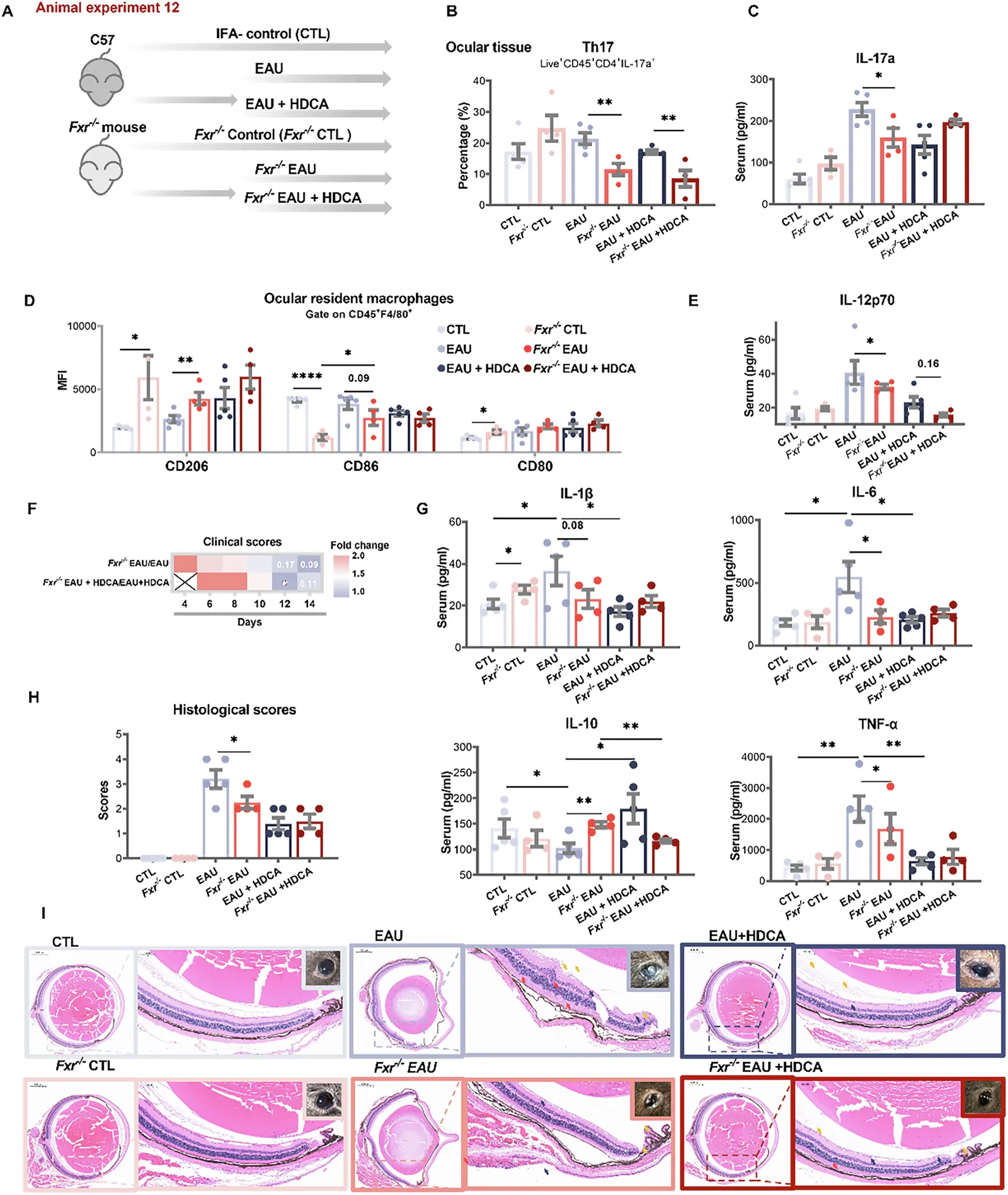
HDCA ameliorates autoimmune uveitis through metabolic-immune crosstalk. **A** C57BL/6J mice vs. FXR^–/–^ C57BL/6J mice: FXR signaling immune-metabolic axis. C57BL/6J and FXR^–/–^ C57BL/6J mice were separately divided into three groups: IFA control group, EAU model, and EAU model with HDCA treatment at 50 mg/kg/day for 14 days (*n* = 5 per group for vehicle mice and *n* = 4 per group for FXR^–/–^ mice). **B** Flow cytometry analysis shows the percentages of ocular Th17 cells. **C** Serum levels of IL-17a. **D** Flow cytometry analysis shows the CD206, CD86, and CD80 expression in ocular macrophages. **E** Serum levels of IL-12p70. **F** Clinical score of the six groups. **G** Serum levels of pro-/ anti-inflammatory cytokines. **H** Histological score of the six groups. **I** H&E staining images of eye sections on day 14 post-immunization exhibit the retinal folds (red arrows), neovascularization (blue arrows), and inflammatory cell infiltration (yellow arrows) across the six groups.

To investigate the role of FXR signaling in HDCA-mediated anti-inflammatory effects, we induced experimental EAU in wild-type and *Fxr*^–/–^ C57BL/6 mice, followed by HDCA or vehicle treatment (Fig. 8A). Consistent with prior findings (Fig. 2), HDCA significantly suppressed Th17 cell generation in ocular tissues, as evidenced by reduced IL-17a production, while simultaneously promoting M2 macrophage polarization through elevated CD206 expression and decreased CD86/CD80 levels; strikingly, these immune responses were completely abolished in *Fxr*^–/–^ mice (Fig. 8B–E). Furthermore, clinical scoring and serum inflammatory cytokine analyses revealed that *Fxr*^–/–^ mice displayed a transient inflammatory peak on day 8 post-induction compared to wild-type controls, but ultimately exhibited accelerated resolution of inflammation, with significantly lower clinical scores than wild-type mice at the experimental endpoint (Fig. 8F, G). Comprehensive ocular evaluations integrating histopathological assessment and H&E-stained tissue analysis confirmed the absence of therapeutic efficacy of HDCA in *Fxr*^–/–^ EAU mice, definitively proving HDCA mediates immunomodulation through FXR-dependent signaling pathways (Fig. 8H, I).

Collectively, HDCA-mediated FXR suppression in RPMs enhances oleic acid production, which orchestrates ocular immunoregulation by simultaneously polarizing tissue-resident macrophages toward a CD206^+^ M2 phenotype and suppressing pathogenic Th17 cell responses, ultimately ameliorating AU pathogenesis.

## Discussion

Our study demonstrates that HDCA reprograms splenic RPMs through FXR inhibition, which activates SREBP1c-dependent FASN to enhance oleic acid production. The RPM-derived oleic acid orchestrates coordinated ocular immunomodulation by simultaneously suppressing pathogenic Th17 cell differentiation and promoting M2 macrophage polarization. This is accompanied by a significant reduction of proinflammatory mediators, including IL-6, IL-1β, TNF-α, Th17-derived IL-17a, and macrophage-produced IL-12p70, collectively establishing a robust anti-inflammatory program.

The spleen contains distinct subsets of tissue-resident macrophages, including RPMs, white pulp macrophages, and marginal zone macrophages, each comprising two subpopulations. Among these subsets, RPMs serve critical physiological functions analogous to hepatic Kupffer cells, including clearance of senescent erythrocytes and platelets coupled with iron recycling [26, 27]. By establishing direct contact with blood during the clearance of aging red blood cells, RPMs can influence distal tissues and cells through secreted metabolites [28]. In the current study, we demonstrate that RPM-derived oleic acid suppresses Th17 responses and induces M2 macrophage polarization (CD206⁺) in ocular tissues, revealing a coordinated mechanism for its distal inflammation activity.

Oleic acid (C18:1n9), an ω9 fatty acid (FA), can be acquired through dietary intake or synthesized via de novo lipogenesis [29]. Alongside mead acid and erucic acid, oleic acid demonstrates significant efficacy in mitigating inflammatory responses across acute and chronic disease models [30]. For instance, in male Wistar mice with hyperlipidemia-induced retinal inflammation, oleic acid suppresses systemic pro-inflammatory cytokines (IL-1β, IL-6, TNF-α) while downregulating retinal inflammatory mediators (BLT-1, EP-1, COX-2) [31]. Beyond oleic acid, evidence suggests that the ω3/ω6 FA ratio also serves as a metabolic regulator for inflammation by governing shared enzymatic and receptor networks [32]. Our data reveal significantly decreased oleic acid concentrations along with diminished ω3/ω6 FA ratios in AU patients and the EAU model, while restoration of these mediators correlates with disease amelioration in mice. Meanwhile, the Bayesian network analysis further demonstrates a causal metabolic cascade linking FA imbalance to inflammatory cytokine production, implicating FA dysregulation as a potential driver of AU pathogenesis.

The localized enrichment of FAs, particularly UFAs, modulates the immune microenvironment and exhibits immunoregulatory effects [33]. For instance, evidence demonstrates that the UFA oleic acid remodels the immune microenvironment to regulate tissue-resident T cell function [34]. While systemic administration of free FAs causes undesirable metabolic disturbances [20, 21], compartmentalized immunometabolic reprogramming offers a targeted alternative by harnessing cell-type-specific expression of FA regulatory receptors. Among these receptors, the FXR constitutes an ideal therapeutic target due to its rare effect on adaptive immune cells and regulation of innate immune cells [35, 36], coupled with the unique capacity of innate immune cells, such as macrophages, to modulate receptors through phagocytic activity. Here, we identify the secondary bile acid HDCA as a potent and spatially restricted FXR antagonist that orchestrates localized immunometabolic reprogramming. Specifically, HDCA-mediated FXR inhibition in RPMs initiates SREBP1-dependent lipid metabolic remodeling, leading to selective production of immunomodulatory ω9 fatty acids, notably oleic acid. Critically, although oleic acid itself possesses intrinsic immunoregulatory properties, our data clearly demonstrate its inability to directly regulate RPM function. HDCA, in contrast, not only expands the RPM population but also facilitates oleic acid-mediated paracrine immunomodulation. This dual mechanism effectively resolves inflammation by concurrently suppressing Th17 cell responses and promoting M2-type macrophage polarization, while circumventing the systemic metabolic complications associated with free FA administration. These mechanistic insights position HDCA as a promising therapeutic candidate for AU and other immune-mediated disorders, revealing a novel spleen-to-eye immunometabolic axis and highlighting the potential of receptor-targeted metabolic modulation.

While our findings demonstrate therapeutic promise, key gaps remain in understanding the systemic modulatory role of HDCA. First, although its precursor chenodeoxycholic acid was observed at reduced levels in EAU, HDCA, as a secondary bile acid, remains critically dependent on gut microbiota for its biotransformation. This metabolic constraint, coupled with the established role of intestinal microbiota in bile acid metabolism, strongly suggests that the gut compartment may play an underappreciated role in EAU pathogenesis, warranting dedicated investigation. Second, bile acids exhibit pleiotropic immunomodulatory potential extending well beyond their classical functions in the gut-liver axis. Their efficient systemic absorption via intestinal lymphatics provides a plausible pathway for influencing extra-intestinal immunity, a mechanism that merits further exploration. Moreover, the reciprocal regulation between HDCA-induced ocular Th17 suppression and M2 macrophage polarization represents another critical knowledge gap. The dynamic crosstalk between these immunomodulatory pathways may fundamentally influence both disease progression and therapeutic efficacy. Finally, although HDCA is generally well-tolerated as an endogenous metabolite, comprehensive safety profiling remains imperative for clinical translation, particularly regarding potential off-target effects.

## Materials and methods

### Clinical cohort

The human studies referenced in this paper were approved by the Ethics Committee (Ethics number: 2020KY037) of Shanghai General Hospital, affiliated with Shanghai Jiao Tong University School of Medicine. All participants provided informed consent by signing a consent form before participating in the study. The study included 90 subjects, consisting of 30 healthy controls and 60 patients diagnosed with autoimmune uveitis according to the criteria established by the International and Chinese Nomenclature Committee [37, 38]. The clinical characteristics of both healthy controls and patients are listed in Table S1. Serum samples were collected from all participants for subsequent analysis.

### Mice

C57BL/6J and BALB/c mice were purchased from the Laboratory Animal Service Centre at the Chinese University of Hong Kong (Hong Kong, China). *Nr1h4*^–/–^ mice (*Fxr*^–/–^ mice, Cat. NO. NM-KO-190328) were purchased from the Shanghai Model Organisms Center (Shanghai, China). Animal experiments were permitted by Hong Kong Baptist University (Ethics number: REC/20-21/0584). Female mice aged 4–8 weeks were utilized for the research. The Hong Kong Baptist University Animal Committee approved all animal procedures. All mice were kept in a controlled environment with appropriate access to food and water (12-h light-dark cycle, humidity maintained at 45 ± 5%, temperature range of 20–24 °C). The blood, spleen, and eyeballs were collected for analysis. The serum was collected by centrifuging blood at 2200 × *g* for 10 min and stored at -80 °C for further study.

### Animal experiment 1

Female C57BL/6J mice (4–6 weeks old, *n* = 5/group) were randomized into healthy controls and EAU groups. The EAU mouse model was induced via immunization with the 300 μg human IRBP651-670 peptide (NovoPro) in 0.2 mL complete Freund’s adjuvant (CFA), along with lipopolysaccharide (LPS, 20 μg/mouse, MedChemExpress) and pertussis toxin (PTX, 50 μg/mouse; Invitrogen) administration. followed by a second identical PTX/LPS dose at 48 h post-immunization. Age-matched healthy female mice served as untreated controls.

In contrast to the traditional mouse model of EAU, additional LPS was employed in the constructed mouse models. This addition was motivated by recent research indicating a connection between AU and specific microbial components. Certain cases of AU have been associated with disruptions in gut microbiota and intestinal mucosal barrier function, resulting in the release of substantial amounts of LPS and other microbiota-derived metabolites into the bloodstream [39, 40]. Furthermore, the mouse model was developed using PTX and *Mycobacterium tuberculosis*, both of which are sourced from microbiota components.

### Animal experiment 2, 3, 6, 7, and 10

Female C57BL/6J (*n* = 5 for control groups; *n* = 6 or 8 for treatment groups) and BALB/c (*n* = 5 for control groups; *n* = 6 for treatment groups) mice aged 4–6 weeks were divided into four experimental groups: IFA-control (injected subcutaneously with 0.2 mL Incomplete Freund’s Adjuvant only), CFA-CTL (injected subcutaneously with 0.2 mL complete Freund’s Adjuvant only), EAU model (the methodology described above), and EAU model with hyodeoxycholate (HDCA, MedChemExpress) treatment at 50 mg/kg/day via gavage following the final immunization for 14 days. The EAU and control groups were orally gavaged with PBS. After the induction of EAU, clinical scores were assessed every 2 days. The serum, spleen, and eyeballs were collected for further analysis.

Uveitis severity was assessed every 48 h using a 0-4 clinical scale evaluating optic disc inflammation, vasculitis, retinal lesions/hemorrhages, and detachment (0: no disease; 4: severe inflammation).

### Animal experiment 4

Female C57BL/6J mice were randomly divided into 2 groups (*n* = 8/group): EAU model and EAU model with oral HDCA at 50 mg/kg/day for 14 days. The spleen was collected for flow cytometry analysis.

### Animal experiment 5 and 8

In this animal experiment, a macrophage-depleted mouse model was established using clodronate liposomes (200 μL/mouse; FormuMax). Specifically, we injected clodronate liposomes intraperitoneally and established the EAU mouse models after 2 days. The injection of clodronate liposomes was repeated on day 5, while control mice received an injection of PBS matched in volume to the clodronate liposomes. The serum and spleen were collected for further analysis.

### Animal experiment 9

Female C57BL/6J mice were randomly divided into three groups (*n* = 6/group): EAU model, EAU model treated with oleic acid (150 mg/kg/day; Macklin) via gavage following the final immunization for 14 days, and the HDCA-treated EAU model (50 mg/kg/day). The spleen, serum, and eyeballs were collected for further analysis.

### Animal experiment 11 and 12

*Fxr*^–/–^ female mice aged 4–8 weeks were randomly divided into three groups, including the *Fxr*^*–/–*^control group (IFA), *Fxr*^*–/–*^ EAU group, and *Fxr*^*–/–*^ EAU model with HDCA treatment at 50 mg/kg/day (*n* = 4/group). All groups are compared with wild-type (C57BL/6J) groups (*n* = 5/group). The spleen, serum, and eyeballs were collected for further analysis.

### Pharmacokinetic studies

C57BL/6J mice (including male and female) were orally gavaged with HDCA (50 mg/kg, *n* = 2/time point) for 0, 0.25, 0.75, 1, 2, 4, 8, 16, 22, and 24 h. Following administration, eyeballs, spleens, and livers were collected for pharmacokinetic studies.

### Cell culture

All Cells were cultured in pyruvate-free DMEM (Gibco) supplemented with 10% heat-inactive FBS (Gibco) and 1% Penicillin, streptomycin, and amphotericin B (Gibco) at 37 °C and 5% CO_2._

This study utilized two types of mouse macrophages: the RAW264.7 cell line (ATCC) and bone marrow-derived macrophages (BMDMs). To generate BMDMs, bone marrow cells were isolated from 4- to 8-week-old C57BL/6J mice. Differentiation was induced as follows: bone marrow was extracted from the femur and flushed with PBS using syringes. The cells were centrifuged at 500 × g for 5 min, and red blood cells were lysed using RBC Lysis Buffer (10X) from Biolegend (USA). After centrifugation and washing, the isolated bone marrow cells were cultured in complete DMEM media and 10 ng/ml recombinant mouse M-CSF obtained from Biolegend (USA) for 7 days.

*Fxr*^*–/–*^ BMDMs were induced from bone marrow cells isolated from *Fxr*^*–/–*^ mice. The spleen of C57BL/6J mice was collected and gently crushed on a cell mesh. It was then rinsed with PBS, and the red cells were lysed to obtain a single-cell suspension. Then, the naïve CD4^+^ and CD8^+^ T cells were isolated using the MojoSort™ mouse naïve CD4^+^ and CD8^+^ T cell Isolation Kit (Biolegend).

### Cell experiment 1

Naïve CD4^+^ or CD8^+^ T cells were indirectly co-cultured with BMDMs treated with LPS (1 μg/mL) with or without HDCA (20 μM) under Th17 (anti-CD3/CD28 beads; Gibco), IL-6 (100 ng/mL; R&D), TGF-β (1 ng/mL; R&D) or cytotoxic T lymphocyte induction conditions (anti-CD3/CD28 beads; Gibco), and IL-2 (50 ng/mL; R&D) for 72 h. Cell samples were collected for flow cytometry analysis (*n* = 3/group).

### Cell experiment 2

Naïve CD4^+^ or CD8^+^ T cells were stimulated with different concentrations of HDCA (10, 20, and 40 μM) under Th17 and cytotoxic T lymphocyte induction conditions for 72 h (*n* = 3/group).

### Cell experiment 3

RAW264.7 cells were treated with LPS (1 μg/mL; throughout cell experiments herein) or LPS with different concentrations of HDCA (5, 10, and 20 μM) for 24 h. or LPS with 40 μM HDCA for 0, 2, 4, 8, 24 h (*n* = 3/group). Cell and media samples were collected for metabolomic analysis.

### Cell experiment 4

Naïve CD4^+^ T cells were treated with different concentrations of oleic acid (50 and 100 μM) under Th17 induction conditions for 72 h (*n* = 3/group).

### Cell experiment 5

BMDMs were stimulated by 100 μM oleic acid only, LPS only, or LPS together with different concentrations of oleic acid (100, 200, and 400 μM) for 24 h (*n* = 3).

### Cell experiment 6

LPS-stimulated BMDMs were treated with or without HDCA (20 μM) for 8 h (*n* = 3/group). Cell samples were collected for qPCR, western blot, and transcriptomic analysis.

### Cell experiment 7 and 8

LPS-stimulated RAW264.8 cells were treated with or without the SREBP1C inhibitor, fatostatin (25 μM, Aladdin), or the FASN inhibitor, orlistat (20 μM, MCE) for 8 h. Cell samples were collected for metabolomics analysis (*n* = 3). Cell samples were collected for qPCR and western blot analysis.

### Cell experiment 9

LPS and HDCA (20 μM) treated BMDMs were stimulated with or without the FXR agonist, GW4064 (10 μM; MedChemExpress, MCE) (*n* = 4/group) for 8 h.

### Cell experiment 10

*Fxr*^–/–^ macrophages were induced through *Fxr*^–/–^bone marrow cells from *Fxr*^–/–^ mice. BMDMs *Fxr*^–/–^ and wild-type mice were stimulated with LPS with or without HDCA (20 μM) for 8 h.

### Metabolomic methodology

#### Targeted metabolomics

Targeted metabolomics analysis was performed on serum, cell, and ocular samples using the Q300 Metabolite Assay Kit (Human Metabolomics Institute, Inc., Shenzhen, Guangdong, China), following a previously published method [15, 16]. Samples were thawed on an ice bath to minimize sample degradation. 20 μL of serum and cell extracts were added to a 96-well plate. Then, the plate was transferred to the Biomek 4000 workstation (Biomek 4000, Beckman Coulter, Inc., Brea, California, USA). Ice-cold methanol with partial internal standards was automatically added to each sample and vortexed vigorously for 5 min. The plate was centrifuged at 4000 g for 30 min. Then, the plate was returned to the workstation. An aliquot of 30 μL supernatant was transferred to a clean 96-well plate, and 20 μL of freshly prepared derivative reagents (200 mM 3-NPH in 75% aqueous methanol and 96 mM EDC-6% pyridine solution in methanol) was added to each well. The plate was sealed, and the derivatization was conducted at 30 °C for 60 min. After derivatization, the plate was lyophilized (Labconco, Kansas City, MO, USA) to dry. Then 400 μL of ice-cold 50% methanol solution was added to resolve the sample, followed by 4000 g centrifugation at 4 °C for 30 min. An aliquot of 135 μL supernatant was transferred to a new 96-well plate with 15 μL internal standards in each well. Serial dilutions of derivatized stock standards were added to the left wells. Finally, the plate was sealed for UPLC-MS analysis.

### Quantitative analysis of fatty acids

Ocular fatty acid analysis was performed through acid hydrolysis (8 M HCl, 70 °C, 40 min) using triundecanoin internal standard (5 mg/mL), followed by lipid extraction with diethyl ether: petroleum ether (3 × 50 mL). Extracted lipids were concentrated and transmethylated with 2% methanolic KOH (80 °C, 15 min) and derivatized with 15% BF₃-methanol (80 °C, 2 min). Fatty acid methyl esters (FAMEs) were extracted in n-heptane and dried over anhydrous Na₂SO₄. GC-FID analysis utilized a 100-m bis-cyanopropyl polysiloxane column (0.25 mm ID, 0.20 μm film thickness) with injector temperature at 250 °C (10:1 split ratio) and FID detector at 260 °C. The temperature program initiated at 125 °C (2 min), ramped at 12 °C/min to 180 °C (6 min), then at 3.5 °C/min to 200 °C (20 min), and finally at 5 °C/min to 230 °C (8 min) with nitrogen carrier gas (1 mL/min) and 1-μL injection volume. Quantitation employed a 37-component FAME standard mix with internal standard calibration, confirming baseline separation of all analytes.

### Quantitative analysis of bile acids

The specific steps of quantitative analysis of bile acids were described in our previous work [15–17].

### Flow cytometry methodology and analysis

#### Sample preparation

The spleen was collected and gently crushed on a cell mesh. It was then rinsed with PBS, and the red cells were lysed to obtain a single-cell suspension.

For the collection of ocular single cells, the eyeballs were gathered and dispersed using DNase I (5 mg/ml, Roche) and Collagenase A (5 mg/ml, Roche) at 37 °C for 15 min. The mixture was then gently passed through a cell mesh to isolate single cells.

All splenic and cultured cells, except for the analysis of splenic dendritic cells, were fixed using FOXP3 Fix/Perm Buffer (Biolegend, USA) for 15 min and then washed with PBS. For ocular Th17 cells analysis, the cells were stained by the Zombie Aqua Fixable Viability (Biolegend), then fixed using FOXP3 Fix/Perm Buffer for 15 min. All these cells were treated with TruStain FcX™ (anti-mouse CD16/32) Antibody (Biolegend, USA) at 0.25 µg per sample in 50 µl volume for 5–10 min on ice and then stained the samples. Macrophages, especially RPM, can phagocytose live/dead dyes, causing false positives and thus significantly reducing the target cells, so the live/dead staining was not stained in the analysis of RPMs and Ocular macrophages.

### Flow cytometry analysis

Samples were added to 50 μL PBS containing a mix of flow cytometry antibodies for 20 min and washed with PBS for further analysis on BD Accuri™ C6 Plus Flow Cytometer or BD FACS Aria™ III Sorter. The antibodies and clone numbers are attached to Table S2.

The splenic immune cells were identified as follows: dendritic cells (CD11c^+^ I-A/I-E^+^), total macrophages (CD11b^+^, F4/80^+^), natural killer cells (CD3^-^ Nkp46^+^), γδ T cells (CD3^+^γδ^+^), CD4^+^ T cells (CD3^+^CD4^+^), and CD8^+^ T cells (CD3^+^CD8^+^). For further analysis of splenic tissue-resident macrophages, the markers of white pulp macrophages are CD45^+^ F4/80^lo^ CD11b^hi^, and RPMs are CD45^+^ F4/80^hi^ CD11b^lo^. For ocular immune cells, ocular resident macrophage is CD45^+^F4/80^+^ (CD86 and CD80 for M1, CD206 for M2), and the marker of Th17 cells is Live^+^CD45^+^CD4^+^IL-17a. For cultured macrophages (CD11b^+^F4/80^+^), CD206. CD86 and CD80 were used as markers of M1 and M2 macrophages. For the analysis of cytotoxic T lymphocytes in CD8 T cells, the marker is CD107a^+^.

The nozzle of flow cytometry is 100 μm, and a 2.0 ND filter was used to identify target cells. Blank unstained control was used to adjust the voltage of the flow cytometry. Single staining controls containing cells and compensation beads (Biolegend, USA) were used to set the compensation for each dye. Fluorescence minus one control was used to gate the positive cells. The data was analyzed via FlowJo. All gate strategies were attached in Fig. S3.

### Transcriptome analysis

BMDMs were treated by LPS with or without HDCA (20 μM) for 8 h and collected for transcriptome analysis. Total RNA was extracted using Trizol Reagent (Takara, Japan) according to the manufacturer’s instructions. RNA samples were evaluated for quality using the A260/A280 absorbance ratio with a Nanodrop ND-2000 system (Thermo Scientific, USA). Subsequently, the RNA Integrity Number (RIN) was determined by employing an Agilent Bioanalyzer 4150 system (Agilent Technologies, CA, USA). Paired-end libraries were prepared using an ABclonal mRNA-seq Lib Prep Kit (ABclonal, China) following the manufacturer’s instructions. The mRNA was purified from 1 μg total RNA using oligo (dT) magnetic beads. The mRNA fragments were then fragmented using divalent cations at elevated temperatures in the ABclonal First Strand Synthesis Reaction Buffer. First-strand cDNAs were synthesized with random hexamer primers and Reverse Transcriptase (RNase H) using mRNA fragments as templates. Subsequently, second-strand cDNA synthesis was carried out using DNA polymerase I, RNase H, buffer, and dNTPs. The synthesized double-stranded cDNA fragments were then adapter-ligated to prepare the paired-end library. Adaptor-ligated cDNA was used for PCR amplification. The PCR products were purified using the AMPure XP system, and the library quality was assessed on an Agilent Bioanalyzer 4150 system. Finally, sequencing was performed with an Illumina Novaseq 6000/MGISEQ-T7 instrument. The Illumina/BGI platform data were utilized for bioinformatics analysis. All analyses were conducted using an in-house pipeline developed by Shanghai Applied Protein Technology.

### Western blot

Antibodies were obtained from reputable suppliers: SREBF1 from Abclonal (A15586), FXR1 from Abcam (ab51970), and β-actin from Santa Cruz (sc-47778).

After stimulation, cells were lysed in RIPA buffer (ThermoFisher), supplemented with 1 mM PMSF (Cell Signaling Technology). Protein lysates were collected by centrifugation at 12,000 x *g* for 15 min at 4 °C. After denaturation, 30 μg proteins in each group were loaded on SDS-PAGE and transferred onto a PVDF membrane (Millipore). Membranes were blocked with 5% fat-free milk diluted with TBST. Then, membranes were incubated overnight with the primary antibody, followed by incubation with the secondary antibody. Finally, bands were visualized with ECL (Bio-Rad) and analyzed using ImageJ software. Protein expression was quantified by measuring band intensity, and full, uncropped Western blot images are available in the Supplementary Information.

### Real-time quantitative PCR

RNAiso Plus extracted the total RNA of culture macrophages according to the manufacturer’s instructions (#9108, Takara, Japan). Thermo Scientific NanoDrop One (Thermo Fisher Scientific, USA) analyzed total RNA concentration. Complementary DNA (cDNA) was generated using PrimeScript™ RT Master Mix (RR036A, Takara, Japan). qPCR was performed in a 5ul reaction volume using TB Green Premix Ex Taq II (RR82WR, Takara, Japan) in 384 wells. The primers for qPCR were synthesized by Sangon Biotech (Shanghai, China) and listed in Table S3. The value of the target genes was normalized to GADPH, and the fold changes to the control group were calculated to determine the relative expression of target genes.

### Cytokines analysis

In this study, serum levels of cytokines, including IL-1β, IL-6, IL-10, IL-17a, IL-12p70, and TNF-α, were analyzed through the DUOSET ELISA kits according to the manufacturer’s instructions (R&D, USA).

### Fundus photography and OCT

For retinal imaging, pupils were dilated with Mydrin-P (0.5% tropicamide and 0.5% phenylephrine; Santen Pharmaceutical) after anesthesia. A physiological solution (Hycell; 2% hydroxypropyl methylcellulose, Sigma) was applied to the cornea, and a coverslip was used to equalize refraction. The retinas were examined for uveitis using fundus imaging and optical coherence tomography (OCT; IIS Science).

### Hematoxylin and eosin (H&E) staining

The mouse eyeballs were fixed with paraformaldehyde, dehydrated, and subsequently embedded in paraffin wax. Slices measuring 6 mm were then obtained and stained with hematoxylin and eosin for proper examination and analysis. Histological images were captured from the standardized central retina and ciliary body regions to ensure consistency across samples. EAU mouse models were evaluated for each eye using a scoring system from 0 to 3: Score 0: No disease present and normal retinal architecture. Score 1: Mild infiltration in the uvea, vitreous humor, and retina, with one small granuloma and no retinal folds or vasculitis. Score 2: Moderate infiltration, along with retinal folds, vasculitis, shallow focal detachments, small granulomas, and localized photoreceptor damage. Score 3: Moderate to severe infiltration, extensive retinal folding, large detachments, subretinal neovascularization, moderate photoreceptor cell damage, and medium-sized granulomatous lesions.

### Immunofluorescence staining for SREBP1 in cultured macrophages

BMDM or *Fxr*^*–/–*^ BMDM were cultured on glass coverslips, fixed with 4% paraformaldehyde (15 min, RT), permeabilized with FOXP3 Fix/Perm Buffer for 15 min, and blocked with 5% BSA/PBS for 1 h. Cells were incubated overnight at 4 °C with rabbit anti-SREBP1 antibody (1:200; Abclonal A15586) diluted in blocking buffer, followed by Alexa Fluor 594-conjugated goat anti-rabbit IgG secondary antibody (1:500; Invitrogen) for 1 h at RT. Nuclear were counterstained with DAPI (1 μg/mL, 5 min), and the photos were acquired from a Leica DM2500.

### Bioinformatics analysis

In Fig. 1, the random forest model was constructed to determine the potential bile acids related to AU. Using a Random Forest package, this model was built on RStudio (RStudio, Boston, MA, USA). Then, the mean decreased accuracy was used to decide the contribution of bile acids in this model. OPLS-DA was conducted on SIMCA software,

In Fig. S6, immune and ω9-related receptor models were constructed using RCS in R Studio through ggplot2, RMS, and plotRCS packages. The specific steps were described in the figure annotation.

In Fig. 4K, to investigate the causal relationships among HDCA treatment, RPMs, and pro-inflammatory cytokines in AU, a Greedy Bayesian Network was constructed using the following variables, disease status, HDCA treatment, fatty acids including the ω3/ω6 fatty acid ratios and oleic acid levels, relative proportions of immune cells (RPMs), and pro- and anti-inflammatory cytokines including IL-1β, IL-6, IL-12p70, IL-10, IL-17a, and TNF-α. The network was constructed by using the *Deal* package in R software, which employs a greedy search algorithm to iteratively score candidate structures based on Bayesian Information Criterion and posterior probabilities.

### Statistical analysis

The study outcomes were presented as means with SEM (standard error of the mean). All plots were generated by GraphPad Prism 9 (GraphPad Software, USA). The graphical abstract and other plots to annotate the experiments were drawn through PowerPoint (Microsoft, USA). The Kolmogorov-Smirnov normality test was utilized to determine the distribution of the sample data. Suppose two groups follow a normal distribution and have homogeneity of variances; an Unpaired one-way Student’s *t* test was used. Otherwise, the Wilcoxon Rank Sum test was used. Potential confounders (sex and age) were adjusted using MCEE (Metabolic Confounding Effect Elimination), a data preprocessing method developed by our group to account for metabolic covariates [41, 42]. The level of significance, denoted by asterisks, indicates the classification of the *p*-value (*P < 0.05, **P < 0.01, ***P < 0.001, and ****P < 0.0001).

## Supplementary information

Supplementary Information_Tables

Supplementary Information_Figures

## Data Availability

All data supporting the results of this study are available in the article and the Extended Data File or upon request from the corresponding author.
