# Supplementary material for: Gut-derived hyodeoxycholate reprograms the spleen–eye immunometabolic axis to suppress autoimmune uveitis
Source: Cell Death Differ. 2026 Mar 11;33(9):1798–813. doi: 10.1038/s41418-026-01696-8 (PMC13545224; doi:10.1038/s41418-026-01696-8)
Supplement: Supplementary file 1 [file 41418_2026_1696_MOESM1_ESM.docx]

**Table S1 Clinical characteristics of the healthy Patients with AU in the clinical cohort**

| **Characteristics** | **Healthy (mean±SE)** | **AU (mean±SE)** |
| --- | --- | --- |
| **Total** | 30 | 60 |
| **Age(years)** | 56.13±17.31 | 47.70±15.70 |
| **gender** |  |  |
| **Male** | 10 | 36 |
| **Female** | 20 | 24 |
| **White blood cells(10^9^/L)** | 5.97±1.33 | 7.43±2.03 |
| **Glucose(mmol/L)** | 5.27±0.42 | 6.1±2.24 |
| **Total triglycerides(mmol/L)** | 1.27±0.67 | 0.95±0.44 |
| **Total cholesterol(mmol/L)** | 5.44±1.18 | 4.81±1.43 |
| **Diagnose*** | - | a (2), b (6), c (9), d (12),  e (17), f (1), g (2), h (3),  i (4), j (1), k (2) |

*a= Behcet syndrome, *b= Vogt–Koyanagi–Harada disease,

*c=Idiopathic acute anterior uveitis, *d=HLA associated acute anterior uveitis, *e=pan uveitis, *f= Juvenile ankylosing spondylitis, *g=Rheumatoid arthritis, *h=Ankylosing spondylitis, *i=Posner-Schlossman Syndrome, *j=Fuchs Syndrome, *k=Scleritis.

*(number) means the number of patients were diagnosed.

**Table S2 Flow cytometry antibodies**

| **Antibodies** | **Source** | **Clone** |
| --- | --- | --- |
| PerCP/Cyanine5.5 anti-mouse CD11c Antibody | Biolegend | N418 |
| APC/Cyanine7 anti-mouse CD45 Antibody | Biolegend | 30-F11 |
| FITC anti-mouse F4/80 Antibody | Biolegend | BM8 |
| PE anti-mouse CD80 Antibody | Biolegend | 16-10A1 |
| PerCP/Cyanine5.5 anti-mouse/human CD11b Antibody | Biolegend | M1/70 |
| APC anti-mouse CD206 (MMR) Antibody | Biolegend | C068C2 |
| FITC anti-mouse CD3 Antibody | Biolegend | 17A2 |
| PE anti-mouse I-A/I-E Antibody | Biolegend | M5/114.15.2 |
| APC anti-mouse CD8a Antibody | Biolegend | 53-6.7 |
| PE/Cyanine7 anti-mouse CD4 Antibody | Biolegend | GK1.5 |
| PerCP/Cyanine5.5 anti-mouse IL-17A Antibody | Biolegend | TC11-18H10.1 |
| PE/Cyanine7 anti-mouse CD8a Antibody | Biolegend | 53-6.7 |
| FITC anti-mouse CD4 Antibody | Biolegend | GK1.5 |
| APC anti-mouse CD107a (LAMP-1) Antibody | Biolegend | 1D4B |
| APC Anti-mouse TCR γ/δ Antibody | Biolegend | GL3 |
| PE/Cyanine7 anti-mouse CD86 Antibody | Biolegend | GL-1 |
| PerCP/Cyanine5.5 anti-mouse CD335 (NKp46) Antibody | Biolegend | 29A1.4 |
| TruStain FcX™ PLUS (anti-mouse CD16/32) Antibody | Biolegend | S17011E |
| Zombie Aqua™ Fixable Viability Kit | Biolegend | - |
| NR1H4 Rabbit Polyclonal Antibody | Biodragon |  |
| SREBF1 Rabbit pAb (A15586) | ABclonal | -- |
| Goat Anti-Rabbit IgG(H&L) [iFluor 647], pAb | Ningyangbui |  |

**Table S3 qPCR primer**

| **Gene name** | **Species** | **Forward primer (5’ to 3’)** | **Reverse primer (5’ to 3’)** |
| --- | --- | --- | --- |
| ***Gadph*** | Mouse | AACTTTGGCATTGTGGAAGG | ACACATTGGGGGTAGGAACA |
| ***Car*** | Mouse | GGAGGACCAGATCTCCCTTC | ATTTCATTGCCACTCCCAAG |
| ***Pparγ*** | Mouse | CCACCAACTTCGGAATCAGCT | TTTGTGGATCCGGCAGTTAAGA |
| ***Vdr*** | Mouse | CACCTGGCTGATCTTGTCAGT | CTGGTCATCAGAGGTGAGGTC |
| ***Tgr5*** | Mouse | TTCTCTCTGTCCGCGTGTTG | GGTGCTGCCCAATGAGATGA |
| ***Fxr*** | Mouse | AGGGGATGAGCTGTGTGTTG | ACACTGGATTTCAGTTAACAAACCT |
| ***Ahr*** | Mouse | CCACTGACGGATGAAGAAGGA | ATCTCGTACAACACAGCCTCTC |
| ***Il-1β*** | Mouse | GAAATGCCACCTTTTGACAGTG | TGGATGCTCTCATCAGGACAG |
| ***Il-6*** | Mouse | CTGCAAGAGACTTCCATCCAG | AGTGGTATAGACAGGTCTGTTGG |
| ***Tnf-α*** | Mouse | CAGGCGGTGCCTATGTCTC | CGATCACCCCGAAGTTCAGTAG |
| ***Il-10*** | Mouse | CGGGAAGACAATAACTGCACCC | CGGTTAGCAGTATGTTGTCCAGC |
| ***Il-12*** | Mouse | CTGCCCCATTGACAAAAGGC | CTAACCGTCGGTCATCAGC |
| ***Pxr*** | Mouse | GACCTGCCTATTGAGGACCA | TTCTGGAAGCCACCATTAGG |
| ***Shp*** | Mouse | GGTCCCAAGGAGTATGCGTA | AGGGCTCCAAGACTTCACAC |
| ***Srebp1c*** | Mouse | GGAGCCATGGATTGCACATT | GGCCCGGGAAGTCACTGT |
| ***Srebp1a*** | Mouse | CAGACACTGGCCGAGATGTG | GAGCTGGAGCATGTCTTCGAT |
| ***Fasn*** | Mouse | GCCCAGACAGAGAAGAGGCA | CTGACTCGGGCAACTTCCC |
