# Supplementary material for: Gut-derived hyodeoxycholate reprograms the spleen–eye immunometabolic axis to suppress autoimmune uveitis
Source: Cell Death Differ. 2026 Mar 11;33(9):1798–813. doi: 10.1038/s41418-026-01696-8 (PMC13545224; doi:10.1038/s41418-026-01696-8)
Supplement: Supplementary file 2 [file 41418_2026_1696_MOESM2_ESM.docx]

**Fig. S1 (A)**, Serum levels of bile acids in clinical cohort. **(B)**, Serum levels of unsaturated fatty acids in clinical cohort. **(C)**, Serum levels of bile acids in animal experiment 1. **(D)**, Serum levels of unsaturated fatty acids in animal experiment 1.

**Fig. S2** **(A)**, Gate strategies for ocular Th17 cells. **(B)**, Gate strategies for ocular macrophages. **(C)**, Clinical scores of IFA-CTL, CFA-CTL, EAU, and HDCA treated EAU BALB/c mice. After the induction of EAU, clinical scores were assessed every two days. **(D)**, Histological scores. **(E)**, Hematoxylin and eosin (H&E) staining images of ocular tissues on day 14 after immunization (retinal folds, red arrows; renewed vasculitis, blue arrows; inflammatory cell infiltration, yellow arrows). **(F)**, Serum levels of pro- and anti-inflammatory cytokines. **(G)**, In this animal experiment, C57BL/6J mice were oral gavage with HDCA (50mg/kg, n=2 per time point) for 0, 0.25, 0.75, 1, 2, 4, 8, 16, 22, and 24 hours. Eyeballs, spleen, and liver were collected for pharmacokinetic analysis.

**Fig. S3 (A)**, Gate strategies for mouse RPM and WPM on BD FACSAria™ III Sorter. **(B)**, Gate strategies for splenic immune cells including dendritic cells (live cells, CD11c^+^ I-A/I-E^+^), natural killer cells (fixed cells, CD3^-^ Nkp46^+^), γδ T cells (fixed cells, CD3^+^ γδ^+^), CD4^+^ T cells (fixed cells, CD3^+^ CD4^+^), and CD8^+^ T cells (fixed cells, CD3^+^ CD8^+^) on BD Accuri™ C6 Plus Flow Cytometer. **(C)**, Flow cytometry analysis for the percentages of splenic immune cells (n=4 per group).

**Fig. S4** Levels of oleic acid in media (cell experiment 3; n=3).

**Fig. S5** Flow cytometry analysis shows the percentage of CD80^+^ macrophages and the mean fluorescence intensity (MFI) of CD80, CD206, and CD86 in LPS-stimulated BMDMs with HDCA treatment (n=3).

**Fig. S6** LPS-stimulated RAW264.7 cells were treated with different concentrations of HDCA (0, 5, 10, and 20 μM; n = 4). Metabolomic analysis, flow cytometry analysis, and qPCR analysis were conducted. Two models including the oleic acid related-receptor model and the Th17-related receptor model, were constructed through the restricted cubic spline (RCS) to determine the potential receptors. In the oleic acid-related receptor model, cellular levels of oleic acid (*Y_oleic acid_*) are the independent variables and the relative expression of farnesoid X receptor (*Fxr*), G protein-coupled bile acid receptor 1 (*Tgr5*), constitutive androstane receptor (*Car*), vitamin D receptor (*Vdr*), aryl hydrocarbon receptor (*Ahr*), retinoid X receptor (*Rxr*), and peroxisome proliferator-activated receptor (*Ppar*); (*X_r_*) is a dependent variable individually. The oleic acid-related receptor model is *Y_oleic acid_* = RCS (*X_r_*). In the Th17-related receptor model, LPS -stimulated RAW264.7 cells treated with different concentrations of HDCA (0, 5, 10, and 20 μM; n = 4) indirectly co-cultured with CD4 T cells under Th17 induction condition. The percentage of Th17 cells was the independent variable (*Y_Th17_*), and the relative expression of receptors is the dependent variable (*X_r_*). The Th17-related receptor model is *Y_th17_* = RCS (*X_r_*). Collectively, the *Fxr* receptor was screened out from these receptors.

**Fig. S7 (A)**, Splenic levels of bile acids (FXR agonist, pink; FXR antagonist, purple). **(B)**, The percentage of RPMs in *Fxr*^-/-^ mice or wild type mice.

**Original western blot 1** Full and uncropped western blots in Fig. 6E

**Original western blot 2** Full and uncropped western blots in Fig. 7E
